# Urea-driven nitrification contributes to N_2_O production in the oligotrophic euphotic ocean

**DOI:** 10.1093/ismejo/wraf281

**Published:** 2025-12-18

**Authors:** Ting Gu, Zhuo Chen, David A Hutchins, Jun Sun

**Affiliations:** Research Centre for Indian Ocean Ecosystem, Tianjin University of Science and Technology, Tianjin 300457, P. R. China; State Key Laboratory of Geomicrobiology and Environmental Changes, China University of Geosciences, Wuhan, Hubei 430074, P. R. China; Research Centre for Indian Ocean Ecosystem, Tianjin University of Science and Technology, Tianjin 300457, P. R. China; State Key Laboratory of Geomicrobiology and Environmental Changes, China University of Geosciences, Wuhan, Hubei 430074, P. R. China; Department of Biological Sciences, University of Southern California, Los Angeles, CA 90089, United States; Research Centre for Indian Ocean Ecosystem, Tianjin University of Science and Technology, Tianjin 300457, P. R. China; State Key Laboratory of Geomicrobiology and Environmental Changes, China University of Geosciences, Wuhan, Hubei 430074, P. R. China

**Keywords:** ammonia-oxidizing archaea, urea oxidation, nitrous oxide, nitrification, ocean acidification, isotopic tracing

## Abstract

Urea is an important alternative nitrogen source to ammonium for nitrification in oligotrophic oceans, yet its role in substrate-driven nitrous oxide (N_2_O) production remains poorly constrained. Here, we combined N_2_O isotopomer profiling, ^15^N-tracer incubations, and metagenomics to quantify and mechanistically resolve substrate-specific archaeal nitrification in the western tropical Pacific euphotic zone. Isotopomer-based mixing and fractionation model indicated that archaeal nitrification accounted for 69.6% ± 14.1% of microbial sources of N_2_O in oxygenated epipelagic waters. Depth-integrated urea-driven nitrification contributed 14%–41% of total nitrification and 21%–39% of nitrification-derived N_2_O, with contributions regulated by substrate proportions. Acidification experiments showed that pH decline inhibited ammonium-driven nitrification (median 21.9%) and enhanced urea oxidation (median 61.9%), whereas N_2_O production increased for both substrates (median 35.9% and 38.0%). In addition, experimental acidification induced opposite shifts in hybrid versus double-labeled N_2_O, suggesting pH-driven shifts N-intermediate chemistry and intracellular partitioning. Metagenomic results support the globally widespread urea-type AOA. Together, these results indicate that urea-driven nitrification constitutes a non-negligible, substrate-dependent source of N_2_O in oligotrophic euphotic zones. We recommend that Earth-system N-cycle models represent urea and ammonium oxidation as distinct pathways with pH-sensitive yields to improve projections of marine nitrification and N_2_O fluxes under acidification.

## Introduction

Nitrogen limits the primary productivity of about half of the world’s oceans and also affects the scale of ocean carbon sinks [1, 2]. In well-oxygenated oceans, nitrification is a central nitrogen cycling process that largely determines the stock of oxidized nitrogen pools [3]. The first step of nitrification is the oxidation of ammonia to nitrite (NO_2_^−^) accompanied by the production of nitrous oxide (N_2_O) by ammonia-oxidizing bacteria (AOB) and ammonia-oxidizing archaea (AOA), which are the dominant ammonia-oxidizing microorganisms in the coastal and open ocean [4, 5], and the second step is the oxidation of NO_2_^−^ by nitrite-oxidizing bacteria (NOB) to NO_3_^−^. However, ammonium concentrations in the open ocean (especially in oligotrophic ecosystems) are typically very low (nmol levels) [6, 7], and ammonia-oxidizing microorganisms in the euphotic zone also compete with phytoplankton for NH_4_^+^ [8]. Paradoxically, the observed abundance of AOA in the open ocean is typically several times higher than NOB, maintains coupled nitrification rates even in sunlit surface waters [5]. This mismatch suggests that AOA utilizes alternative nitrogen substrates beyond NH_4_^+^ to sustain nitrification.

Numerous studies have demonstrated nitrification can utilize alternative nitrogen sources besides ammonium, including urea (and cyanate) [9, 10]. Genome-resolved analyses and culture experiments demonstrate that subgroups of AOA and AOB encode urea transporters (UT) and urease, enabling direct hydrolysis of urea to NH_3_ and CO_2_ and subsequent ammonia oxidation, indicating that urea can sustain the energy metabolism of ammonia-oxidizing microbes [4, 10, 11]. Urea can also indirectly fuel nitrification when heterotrophs hydrolyze urea and release NH_4_^+^ to the bulk water [12–14]. The relative importance of these two pathways likely varies with substrate balance and community composition [11, 15]. Recent euphotic-zone incubations indicate that urea accounts for ~20%–36% of nitrite production, mostly via the direct pathway [16]. Whether urea oxidation also drives N_2_O production remains underexplored in the open ocean. Previous *in situ* observation (eutrophic Chesapeake Bay) found lower urea-driven N_2_O production rates but similar yields relative to ammonium oxidation [17], patterns that may not directly expand to AOA-dominated oligotrophic systems [4, 5]. Because urea utilization by ammonia-oxidizing microorganisms requires an energetic investment in the synthesis of urea transporter proteins, ureases, and accessory proteins. This represents a significant cost for autotrophic ammonia-oxidizing microorganisms, implying that ammonia-oxidizing microorganisms in ammonium-enriched environments may forgo utilization of energetically expensive urea [9, 18]. At present, the biochemical pathways for archaeal ammonia oxidation and N_2_O formation remain incompletely resolved. Recent suggests that N_2_O production in the ammonia oxidation pathway may be linked to intermediates such as hydroxylamine (NH_2_OH), NO, and nitroxyl (HNO) [19–21]. Given the pH sensitivity of these nitrogen intermediates, pH shifts are expected to modulate the balance between enzymatic and non-enzymatic N_2_O formation [19, 22].

Here, we hypothesize that AOA in oligotrophic oceans directly oxidize urea, analogous to ammonium oxidation, and that pH modulates the relative pathway contributions via substrate-specific constraints. We tested this with ^15^N tracers (NH_4_^+^, urea) under controlled acidification in the western tropical Pacific (WTP), integrates isotopic geochemistry and molecular biology approaches. This framework enabled us to apportion ammonium- vs urea-driven nitrification to euphotic zone N_2_O production, link pathways to taxa/genes, and resolve pH-dependent shifts in mechanisms. Our results indicate that AOA-mediated urea oxidation constitutes a non-negligible, substrate-dependent source of N_2_O in oligotrophic waters, with implications for nitrogen-cycle and N_2_O prediction under acidification.

## Materials and methods

### On-board incubation

For details on the study area and field sampling (Supplementary methods). Seawater for on-board bottle incubations with chemical measurements was collected from the same Niskin bottle. All incubation bottles were rinsed with 10% HCl, deionized water and sampled seawater before sampling. For profiles of nitrification rates and N_2_O production rates with ammonium and urea as substrates, triplicate 160 ml serum bottles (butyl stoppers; pre-rinsed) with 1-mm glass beads were filled with *in situ* water [23]. Before starting the incubation, 1 ml ^15^NH_4_^+^ or ^15^N-urea (99% of ^15^N atom, Cambridge Isotope) carrier was added to the incubation bottles to make the final tracer concentration 0.5 μmol N L^−1^. The final tracer concentration was typically higher than the *in situ* substrate concentration, accounting for the ^15^NH_4_^+^ and ^15^N-urea labeling incubations final substrate pools 89.0% ± 5.6% and 90.3% ± 7.8%, respectively. Incubations were performed for depths within 0–100 m. Pressure was not simulated and light was excluded by design to suppress photosynthesis, therefore the nitrification and N_2_O production rates are considered as potential rates. Given the different pH sensitivities of ammonium- and urea-based pathways [24, 25], we added an additional acidification treatment with target -ΔpH = 0.2–0.5 units were chosen to simulate end-of-century projections for surface oceans [26, 27]. For the acidification treatment, the pH decreases of 0.2–0.3 was modified by adding 0.5 μmol L^−1^ HCl and 200 μmol L^−1^ NaHCO_3_ solution to the incubation bottles before adding the ^15^N tracer and pre-incubated for 6 h in the dark incubator at *in-situ* temperature (±1°C). At the end of the preincubation, samples were added with ^15^N tracer and immediately filtered through a 0.2 μm syringe filter for 1 bottle of samples and stored at −20°C for initial condition analysis of nitrification rate, and another bottle was added to 200 μl of saturated HgCl_2_ solution for initial N_2_O isotope analysis. The other samples were incubated in a dark incubator close to the *in-situ* temperature (±1°C) for 24 h. At the end of the incubation, samples for nitrification rate measurements were filtered through 0.2 μm syringe filters and stored at −20°C, and samples for N_2_O isotope measurements were immediately added to 200 μl of saturated HgCl_2_ solution and stored at 4°C. In addition, an additional 8 L of seawater was dispensed into 2 polycarbonate Nalgene bottles (only 100 m depth seawater), one of which was filtered with a 0.2 μm filter (Millipore Isopore) before incubation, and the other was acidified by adding the 0.5 μmol L^−1^ HCl and NaHCO_3_ solution, and incubated under the same conditions for 24 h and then filtered with 0.2 μm filter (Millipore Isopore), and the filter membrane samples were stored in liquid nitrogen for following high-throughput sequencing and quantitative polymerase chain reaction (qPCR) analysis.

### Nitrous oxide concentration measurement

N_2_O concentrations were measured using the static headspace injection combined with a gas chromatograph (Agilent GC8860 model equipped with a microelectronic capture detector). The concentrations of the standard gasses (N_2_O/N_2_) were 0.10–3 ppmv (Research Institute of China National Standard Materials), respectively. Dissolved N_2_O concentrations were calculated from headspace measurements using temperature- and salinity-dependent N_2_O solubility following Weiss & Price (1980) [28], results are reported as nmol N_2_O L^−1^. The detection limit of the method was 1.0 nmol L^−1^, and the precision was ~2%.

### Isotopic analyses of nitrous oxide and nitrogen oxides

Isotope workflows used in this study comprised natural abundance N_2_O isotopomers (δ^15^N^bulk^-N_2_O, δ^18^O-N_2_O, and SP) and ^15^N-tracer incubations analyzed. N_2_O isotopomers measured on unamended seawater for pathway apportionment and air-sea mixing correction, N_2_O isotope ratio used to derive potential rates and label partitioning (hybrid ^45^N_2_O vs double-labeled ^46^N_2_O). Isotopomer analysis was not applied to tracer bottles. Dissolved N_2_O isotopomers abundance was analyzed using a modified headspace equilibrium method. The N_2_O gas was delivered to Tracegas through the autosampler; N_2_O gas was then extracted, purified, and trapped by Tracegas, and the nitrogen and oxygen isotope concentration was finally measured using the gas chromatography–isotope ratio mass spectrometer (IRMS; Delta V Plus-Precon, Germany). Nitrogen isotopes at specific sites in N_2_O were analyzed using a modified ion detector while monitoring five Faraday cups at m/z 30, 31 (used for the determination of δ^15^N^α^) and 44, 45, 46 (used for the determination of δ^15^N^bulk^ and δ^18^O) in order to calculate the isotopic fractions of N_2_O by mass analysis of N_2_O (NO^+^) fragment ions containing N atoms in the center of the N_2_O molecule [29, 30]. Two-point calibration of isotope measurement results using international standard N_2_O gas (USGS51 and USGS52). Site preference (SP) is calculated from the difference in δ^15^N between the central (α) and outer (β) N atoms in the linear, asymmetrical N_2_O molecule (NNO) [31]:


(1) \begin{equation*} SP={\delta}^{15}{N}^{\alpha }-{\delta}^{15}{N}^{\beta } \end{equation*}

The analytical precision of δ^15^N^bulk^, δ^18^O, and SP were below 0.2, 0.5, and 1.0‰, respectively.

Concentrations of ^45^N_2_O and ^46^N_2_O in isotope tracer incubation experiments were also analyzed using the modified headspace equilibrium method. Headspace gas was introduced into a gas chromatography–IRMS (Isoprime100, Cheadle, UK) and N_2_O concentrations were determined from ion peak area (m/z = 44) and calibrated using a standard gas of N_2_O/He for calculating N_2_O concentrations from the ion peak area, which was run at ten sample intervals. The accuracy of the method is estimated to be better than ±3% [32].

For the determination of δ^15^N-NO_x_^−^. δ^15^N-NO_x_^−^, and δ^18^O-NO_x_^−^ were obtained using the denitrifier method to convert NO_x_^−^ to N_2_O, detected using a continuous flow IRMS (Isoprime100, Cheadle, UK). The calibration standards for δ^15^N and δ^18^O for NO_x_^−^ detected in this study were USGS32, USGS34, and USGS35.The average precision of sample analysis was ±0.25‰ for δ^15^N-NO_x_^−^ and ± 0.50‰ for δ^18^O-NO_x_^−^.

### Keeling plot analysis and two-endmember mixing model for isotopic correction

To correct for the influence of atmospheric exchange on N_2_O isotopic signatures in surface waters, we applied Keeling plot analyses to estimate the *in situ* isotopic composition of microbially produced N_2_O. This approach assumes that the observed isotopic signature (δ^15^N^bulk^-N_2_O, δ^18^O-N_2_O, and SP) indicate a simple two-endmember mixing between atmospheric N_2_O and N_2_O produced by microbial processes *in situ*. The isotopic composition of the microbially derived endmember can then be inferred as the y-intercept of a linear regression between the isotopic signature and the inverse of the N_2_O concentration (Eq. 2–3).


(2) \begin{equation*} {\displaystyle \begin{array}{c}{\left[{N}_2O\right]}_{obs}={\left[{N}_2O\right]}_{atm}+{\left[{N}_2O\right]}_{micro}\end{array}} \end{equation*}

(3) \begin{equation*} {\displaystyle \begin{array}{c}{\delta}_{obs}\times{\left[{N}_2O\right]}_{obs}={\delta}_{atm}\times{\left[{N}_2O\right]}_{atm}+{\delta}_{micro}\times{\left[{N}_2O\right]}_{micro}\end{array}} \end{equation*}

where [N_2_O] denotes the concentration of N_2_O (in nmol L^−1^), and δ represents its isotopic composition-either δ^15^N^bulk^-N_2_O, δ^18^O-N_2_O or SP. The subscripts “atm” and “micro” refer to the respective sources of the observed N_2_O isotopic signal. Under the assumption that microbially produced N_2_O has a constant isotopic signature throughout the water column, the y-intercept from this regression represents δ_micro_.

### Monte Carlo model for evaluation of the stable isotope mixing and fractionation

We apportioned N_2_O sources and reduction using a Bayesian MCMC framework that jointly fits δ^18^O-N_2_O, δ^15^N^bulk^-N_2_O, and SP. The model considers four pathways, archaeal nitrification (aN), bacterial nitrification (bN), nitrifier denitrification (nD), and denitrification (bD) [33, 34]. The isotopic signature of each source was established in previous pure culture and *in situ* observational studies [35] (Supplementary Table S2), described by closed system dynamics with Rayleigh-type equations:


(4) \begin{equation*} {\displaystyle \begin{array}{c}F={f}_{bN}\bullet{E}_{bN}+{f}_{aN}\bullet{E}_{aN}+{f}_{bD}\bullet{E}_{bD}+{f}_{nD}\bullet{E}_{nD}+\mu \bullet \mathit{\ln}(r)\end{array}} \end{equation*}

where f is the respective fraction of pathway contributing to the N_2_O production and Ei is the end member value of this pathway, r is the residual unreduced N_2_O fraction and μ is the stable isotope fractionation factor associated with N_2_O reduction. F stands for the final N_2_O isotopic signature after mixing of all sources and partial N_2_O reduction. Model analysis was performed using FRAME software (malewick.github.io/frame).

### Nitrification rate and nitrous oxide production rate calculation

Ammonium and urea-driven nitrification rates were determined as the amount of ammonia (^15^NH_4_^+^) or urea (^15^N-urea) oxidized to nitrite (^15^NO_2_^−^) and nitrate (^15^NO_3_^−^).


(5) \begin{equation*} {\displaystyle \begin{array}{c}{R}_{nitr}=\frac{1}{\Delta T}\bullet \frac{C_t-{C}_0}{F_{substrate}}\times 24\end{array}} \end{equation*}

where R_nitr_ is the nitrification rate for ^15^NH_4_^+^ or ^15^N-urea enrichment (nmol N L^−1^ d^−1^). C_0_ and C_t_ are the ^15^NO_x_^−^ concentrations (nmol N L^−1^) at the beginning and end of the incubation, respectively, and F_substrater_ is the percentage of ^15^N atoms in the NH_4_^+^ or urea pool at the beginning of the incubation. ΔT is the incubation time (h).

The N_2_O production of ^15^N-labeled substrates was quantified as the increase in ^45^N_2_O and ^46^N_2_O relative to natural abundance at the end of the incubation. N_2_O production rates (R_N2O_, nmol L^−1^ d^−1^) were calculated according to the following:


(6) \begin{equation*} {\displaystyle \begin{array}{c}{R}_{N2O}=\frac{1}{\Delta T}\bullet \frac{\Delta{C}_{45}+2\bullet \Delta{C}_{46}}{F_{substrate}}\end{array}} \end{equation*}

where ΔC_45_ and ΔC_46_ represent the masses ^45^N_2_O and ^46^N_2_O concentration difference between the beginning and end of incubation. F_substrater_ is the percentage of ^15^N atoms in the NH_4_^+^ or urea pool at the beginning of the incubation. ΔT is the incubation time (h). Based on three times the standard deviation as a reliable enrichment of ^15^N in the product pool, we calculated the detection limit of 0.015 nmol N L^−1^ d^−1^ for nitrification rate and 0.25 pmol N_2_O L^−1^ d^−1^ for N_2_O production rate.

N_2_O yield is defined as the N_2_O production rate as a proportion of the total nitrogen conversion rate (nitrification rate). We estimated the N_2_O yield in ammonium or urea-driven nitrification using the equation.


(7) \begin{equation*} {\displaystyle \begin{array}{c}{N}_2{O}_{yield}=\frac{R_{N2O}}{R_{N2O}+{R}_{nitr}}\end{array}} \end{equation*}

All rates quantify oxidation pathways based on the transfer of ^15^N from substrates to NO_x_^−^ and N_2_O; therefore, ^15^N incorporated into biomass (assimilation) is not counted as production. Incubations were conducted in the dark at *in-situ* temperature with identical tracer levels between control and treatments (final substrate ≈ 0.5 μmol N L^−1^), making treatment contrasts internally comparable even if assimilation competes for substrates.

### Deoxyribonucleic acid extractions and sequencing

DNA samples for metagenomics sequencing were extracted using the DNeasyPowerWater kit (Qiagen, Hilden, Germany). Extracted DNA samples were sent to Magigene gene for metagenomic sequencing, and libraries were prepared for each sample without any amplification steps. Metagenomic sequencing was performed on the NovaSeq 6000 System (Illumina), generating 2 × 150 bp paired-end reads, which were then processed using Fastp (version 0.23.4) to trim low-quality sequences and reads containing ambiguous N bases, Detailed metagenomic analysis methods are provided (Supplementary methods). The control treatment and acidification treatment DNA samples were amplified using universal primers 515F and 806R for the prokaryotic 16S rRNA gene (V4 region) [36] (Supplementary Table S9). PCR products were purified using an agarose gel DNA purification kit (Qiagen, USA) and then sequenced with a MiSeq System (Illumina; Magigene Biological Technology Co. Ltd., Guangzhou, China). We used the DADA2 (v. 1.26.0) pipeline to process the raw sequences into ASVs [37]. The denoised reads were merged using the function *mergePair* and chimeric overlaps were removed with *removeBimeraDenovo*. Taxonomic assignment of ASVs from 16S rRNA gene (V4) amplicons was performed in DADA2 using the SILVA v138.1 reference database.

### Quantitative polymerase chain reaction

Quantification of archaeal *amoA*, bacterial *amoA*, and Archaeal *ureC* gene copies was performed on the Pharmaceutical Analytics QuantStudio 5 Real-Time PCR Detection System (Applied Biosystems) using SYBR Green assay (Supplementary Table S9). The standard quality plasmid was diluted to 1 × 10^9^, and then 10 μl was taken and added to 90 μl of sterile water, and then 10-fold gradient dilution was made to obtain 1 × 10^8^, 1 × 10^7^, 1 × 10^6^, 1 × 10^5^, 1 × 10^4^, 1 × 10^3^, a total of 7 concentration gradients to prepare the standard curve. Each reaction (10 μl) consisted of 5 μl ChamQ SYBR qPCR Master Mix (Applied Biosystems), 0.25 μl of forward and reverse primers, 2 μl of DNA template, and 2.5 μl of molecular biology grade water (Fisher BioReagent). The qPCR procedure consisted of 95°C for 30 seconds followed by 40 cycles of 95°C for 10 seconds, 55°C for 30 seconds, and 72°C for 30 seconds. All amplification efficiencies ranged from 95.6%–100% and the standard curve *R*^2^ ranged from 0.990 to 1.000.

### Statistical analysis

The dataset was analyzed in R (v 4.3.1) using phyloseq (version 1.46.0) [38], vegan (version 2.6.6), and visualized using ggplot2 (version 3.4.3). Alpha diversity analyses were assessed using Shannon and observed ASVs indices and paired sample t-tests to determine if differences between control and acidification treatment groups were significant. Beta diversity analysis was assessed by permutation multivariate analysis of variance (PERMANOVA) and Bray-Curtis distance-based assessment to examine community differences between sample groups. The significance level for both analyses was set at *P* < .05. Correlations between the nitrifying microbial relative abundance and the nitrification and N_2_O production rates were examined using the Person parametric correlation test (*P* < .05). The Sparcc-based microbial co-occurrence network analysis was performed in the “SpiecEasi” (version 1.1.3) software package [39], where sparse correlations between ASVs were calculated by 99 iterations and pseudo *P* values (two sides) were estimated using 100 bootstraps. Finally, edges with absolute values of correlation >0.7 and *P* < .05 were used to construct the network and visualization was done using Gephi (version 0.10). For acidification-control comparisons we summarized percent changes using median (IQR) after censoring rates ≤ LOD. Robustness was evaluated by leave-one-station-out recalculation of medians, bootstrap (10 000 resamples) 95% CIs for medians for paired shifts.

## Results and discussion

### Source of nitrous oxide in the euphotic zone

**Figure 1:**
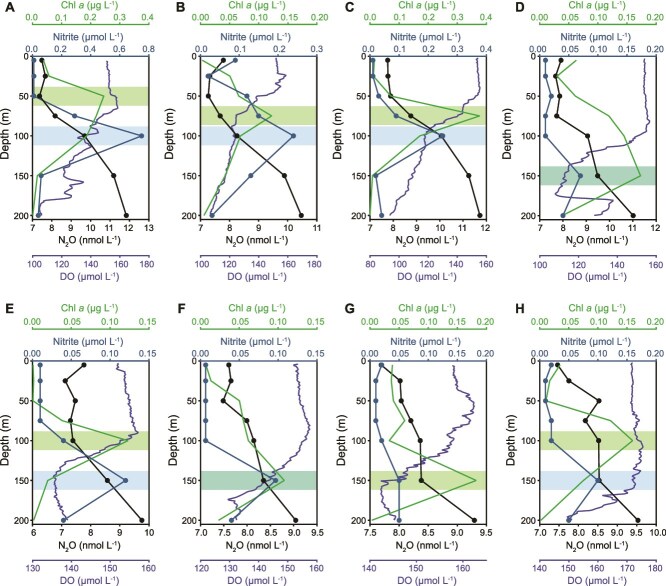
Depth profiles of N_2_O concentration, DO, NO_2_^−^ and chlorophyll *a* at the study stations. (A–H) N_2_O concentration (black dots), DO (solid blue line), NO_2_^−^ concentration (celadon dots) and chlorophyll *a* (solid green line) at stations F01 (A), F02 (B), E01 (C), E02 (D), E03 (E), E04 (F), E05 (G), and E06 (H). Blue bars mark the depth range of the PNM layer, green bars mark the depth range of the DCM. The red dotted line represents the mixed layer depths.

During the WTP research cruise, surface to 100 m depths were sampled at eight stations (details in Methods; Supplementary Fig. 1 and Table S1). Ammonium and urea concentrations in WTP were both low at all depths (NH_4_^+^: 0.03–0.37 μmol N L^−1^; Urea: 0.004–0.36 μmol N L^−1^), which is characteristic of typical oligotrophic oceans [40, 41]. N_2_O concentrations in surface water area were 7–8 nmol N_2_O L^−1^ at all stations and increased with depth (Fig. 1). The vertical distribution pattern of N_2_O showed an opposite trend to dissolved oxygen (DO), and the primary nitrite maximum (PNM) is typically lower than the deep chlorophyll maximum (DCM) layer (Fig. 1). Furthermore, rapid increases in N_2_O were observed in the PNM layer, indicating a strong spatial coupling between N_2_O accumulation and nitrite. Increases in nitrite were also found to be associated with high ammonia oxidation rates [40, 42], these patterns are consistent with an *in situ* nitrification contribution to the observed N_2_O accumulation.

**Figure 2:**
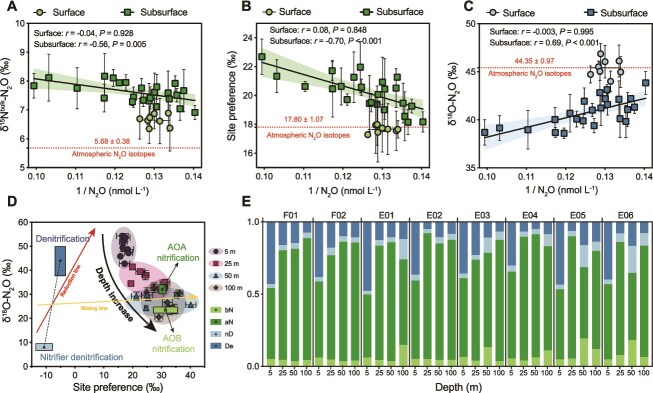
N_2_O isotopomers and source partitioning of N_2_O in surface and subsurface waters of the tropical Western Pacific. (A-C) Keeling plots for δ^15^N^bulk^-N_2_O (A), SP (B), and δ^18^O-N_2_O (C) versus the inverse of N_2_O concentrations (1/[N_2_O]) in surface (circles) and subsurface waters (squares). Shaded bands represent 95% confidence intervals of linear regression. Dashed red lines indicate isotopic endmembers of atmospheric N_2_O, atmospheric isotope values were obtained from previously published work [78]. Y-intercepts reflect microbial N_2_O isotopomers. (D) Atmospheric-corrected microbial N_2_O in SP/O plot, overlaid with reference ranges of microbial processes including AOA and AOB nitrification, nitrifier-denitrification (nD), and canonical denitrification. Given the well-oxygenated conditions at all stations, the “De” field is shown as a reference endmember and likely represents deep-sourced N_2_O mixed rather than *in situ* denitrification. (E) Relative contributions of modeled N_2_O sources across stations and depths, estimated by stable isotope mixing and fractionation model (FRAME). Colors represent contributions from four microbial pathways: bN, aN, nD, and De, across depths of 5, 25, 50, and 100 m.

To investigate the origin of N_2_O, we measured δ^15^N^bulk^-N_2_O, δ^18^O-N_2_O, and SP across depth gradients. These isotopic tracers varied with N_2_O concentrations, particularly in subsurface waters (25–100 m) (Supplementary Fig. 2). Keeling plots (1/[N_2_O] vs isotope values) revealed significant linear relationships for δ^15^N^bulk^-N_2_O, δ^18^O-N_2_O, and SP in the subsurface, with Pearson r = −0.56, 0.69, and − 0.70 and all *P* < .01, respectively. No significant relationships were observed in surface layers (all *P* > .05) (Fig. 2A–C). These results indicate that microbial production dominates at depths below the surface, whereas surface water shows atmospheric sources of N_2_O.

We corrected atmospheric mixing using a dual-endmember correction (Equations 2–3), yielding microbial δ^15^N^bulk^-N_2_O, δ^18^O-N_2_O, and SP values [43]. The corrected isotopic cluster near AOA-nitrification isotopic fingerprints and converge with depth (Fig. 2D) [44]. We applied a stable isotope mixing and fractionation model (FRAME) using δ^15^N^bulk^-N_2_O, δ^18^O-N_2_O, and SP jointly to apportion pathways [45] (Fig. 2E and Supplemental Table S2). Model results confirmed that AOA-mediated nitrification accounted for the majority of N_2_O production in the euphotic zone (mean 69.6% ± 14.1%) (Fig. 2E and Supplemental Table S3), consistent with previous results in oligotrophic waters [46–48]. Given that all stations were well-oxygenated, *in situ* denitrification is unlikely to be the primary source of N_2_O in our photic zone samples. The contribution from denitrification may reflect N_2_O transported to the surface from deep sources rather than local denitrification [49].

### Nitrification rates and nitrous oxide production rates driven by different substrates

**Figure 3:**
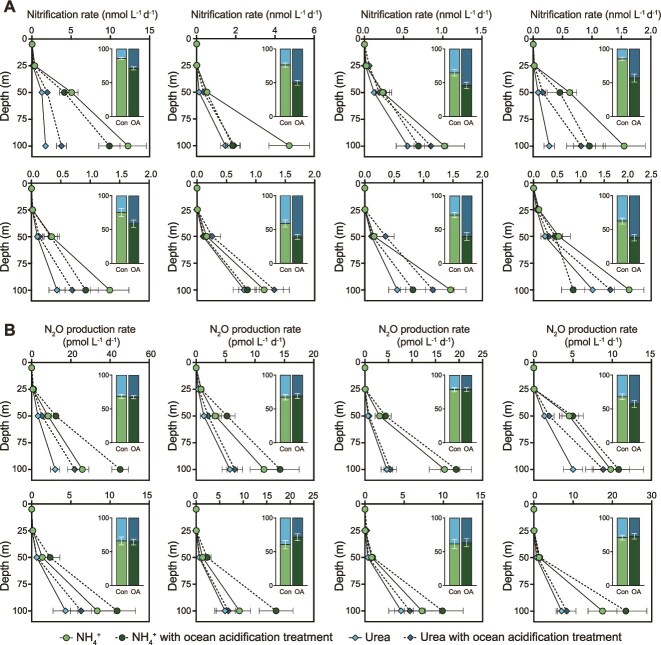
Depth profiles of substrate-specific nitrification and N_2_O production rate. (A) Nitrification rates (nmol L^−1^ d^−1^). (B) N_2_O production rates (pmol L^−1^ d^−1^). Each small panel shows a station (0–100 m). Green circles represent NH_4_^+^-driven rate (control pH); dark green circles represent NH_4_^+^-driven rate under OA treatment (ΔpH ≈ −0.2 to −0.3); blue diamonds represent urea-driven rate (control); dark blue diamonds represent urea-driven rate under OA. Error bars denote ± SD (*n* = 3 bottles). Rates are potential rates (see Methods). Insets plot shows the contribution ratio of ammonium and urea to the depth-integrated total rate under different treatments, error bars show mean ± SD.

We measured potential depth profiles (0–100 m) of ammonia- and urea-driven nitrification (R_AN_, R_UN_) and N_2_O production (R_AN-N2O_ and R_UN-N2O_) at eight stations. Ammonia and urea oxidation rate depth profiles at all stations showed a similar vertical pattern, with a gradual increase in rates from the surface to 100 m (Fig. 3A). Maximum ammonia oxidation rates (1.02–12.26 nmol L^−1^ d^−1^, mean 3.19 nmol L^−1^ d^−1^) were typically higher than maximum urea oxidation rates (0.27–1.74 nmol L^−1^ d^−1^, mean 0.89 nmol L^−1^ d^−1^) (Fig. 3A). Depth-integrated urea-driven nitrification rates contributed 14.2%–40.7% (median 27.4%, IQR: 21.4%–35.4%) of the total ammonia and urea nitrification rates, and depth-integrated urea R_UN-N2O_ accounted for 20.6%–38.8% (median 31.6%, IQR: 29.8%–34.6%) of the total ammonia and urea N_2_O production rates, indicating that urea-driven nitrification makes a non-negligible (one-quarter to one-third) contribution to the production of NO_x_^−^ and N_2_O in oligotrophic oceans (Fig. 3B). In addition, depth-integrated nitrification rates were positively correlated with primary productivity (R_AN_, *P* = .04, R_UN_, *P* = .02; Supplementary Fig. 3), indicating the high primary productivity provided organic matter for microbial decomposed into NH_4_^+^ and urea [50].

We examined how the ambient substrate balance shapes pathway partitioning. Across stations and depths, ammonium-driven nitrification positively with NH_4_^+^ (slope = 23.47, *R*^2^ = 0.70, *P* < .001), and the associated N_2_O production also increased with NH_4_^+^ (slope = 47.51, *R*^2^ = 0.43, *P* < .001) (Supplementary Fig. 4A). Urea-driven nitrification and N_2_O production also showed significant linear relationship with bulk urea (both *P* < .05; Supplementary Fig. 4), consistent with observations in the Chesapeake Bay [51]. The ratio of urea:NH_4_^+^ in bulk waters positively with the relative contribution of the urea pathway (slope = 0.24, *R*^2^ = 0.23, *P* = .031) and the N_2_O production ratio (slope = 0.32, *R*^2^ = 0.33, *P* = .008) (Supplementary Fig. 4). Together, these results show that when urea becomes relatively more available than ammonium, the system shifts toward urea-driven nitrification and N_2_O production, supporting a substrate-specific response.

### Unique response of nitrification rates and nitrous oxide production rate of different substrates to ocean acidification

To probe urea-driven nitrification pathways given the different pH sensitivities of NH_4_^+^ and urea [52], we applied additional acidification treatment (ΔpH = 0.22 ± 0.03). Ammonium- and urea-driven nitrification responded divergently under this treatment (Fig. 3 and Supplementary Figs 5 and 6). In addition, pH decreases reduced ammonium-driven nitrification by a median 21.9% (IQR: 11.9%–32.0%) but enhanced urea-driven nitrification by a median 61.9% (IQR: 46.1%–125.5%). In contrast to the unique effects of acidification treatments on nitrification rates, both ammonium- and urea-driven N_2_O production rates showed positive feedbacks with decreasing pH (R_AN-N2O_: median 35.9%, IQR: 14.5%–54.2%; R_UN-N2O_: median 38.0%, IQR: 16.5%–48.1%, Supplementary Fig. 7). The inhibitory effect of acidification on nitrification rates is consistent with previous observations in the open ocean [24, 25] and the meta-analysis results (significant negative effect of acidification on ammonia oxidation ranging from 34% ± 10%) [53].

Ocean acidification (OA) typically depresses ammonia-based nitrification [24, 25]. Largely availability of NH_3_ declined exponentially with pH decrease (NH_3_ + H^+^ ↔ NH_4_^+^; pKa = 9.25) [54]. This has also been used to describe the inhibition of nitrification rates observed in other ocean regions under pH decline [24, 25, 55]. In addition, pH decrease leads to the accumulation of toxic intermediates such as NO and NO_2_^−^, which would have a negative effect on nitrifying microorganisms [56, 57]. By contrast, whether urea-driven nitrification is pH-dependent remains unclear, soil studies suggest that lower free NH_3_ can promote urea use by ammonia oxidizers [58, 59], and several AOA cultures show broad pH tolerance with maximal ammonium-based growth at pH < 7 [10, 60]. qPCR showed AOA abundance increased under acidification (Wilcoxon test, *P* = .004, Supplementary Fig. 8). Across stations, archaeal *amoA* gene correlated with NH_4_^+^-driven nitrification (slope = 0.23, *R*^2^ = 0.38, *P* = .01) and NH_4_^+^-driven N_2_O production (slope = 0.08, *R*^2^ = 0.32, *P* = .02) (Supplementary Fig. 9A and B), linking higher AOA abundance to stronger NH_4_^+^ oxidation and N_2_O production. Thaumarchaeal *ureC* coupled with urea-driven nitrification (slope = 0.90, *R*^2^ = 0.34, *P* = .02) and urea-driven N_2_O production (slope = 0.18, *R*^2^ = 0.31, *P* = .03) (Supplementary Fig. 9E), and the Thau-*ureC*: *amoA* ratio tracked the urea:NH_4_^+^ oxidation ratio (slope = 2.10, *R*^2^ = 0.35, *P* = .03) (Supplementary Fig. 9C), indicating that AOA harboring *ureC* make a significant contribution to N_2_O production during urea oxidation [51, 61]. By contrast, the N_2_O (urea:NH_4_^+^) ratio did not related with Thau-*ureC*: *amoA* (*P* = .47; Supplementary Fig. 9F), consistent with N_2_O yield being additionally controlled by pH-sensitive intermediate chemistry (e.g. NH_2_OH/NO/HNO coupling) rather than community composition alone. Together, these lines of evidence show that AOA remain active under acidification and that urea-type AOA are widespread and contribute measurably to nitrification and N_2_O formation in the western Pacific ocean.

OA also altered ammonium- and urea-driven N_2_O yields, with ammonium-driven N_2_O yields increasing from 0.56% ± 0.40% to 1.23% ± 1.18%, and urea-driven N_2_O yields decreasing from 0.72% ± 0.42% to 0.57% ± 0.46% (Supplementary Figs 10 and 11). In addition, hybrid N_2_O (i.e. from one ^15^NH_4_^+^ and one unlabeled nitrogen (N) source, e.g. NO_2_^−^, NO) was the primary pathway for N_2_O production in nitrification, whatever the substrate and pH conditions (Supplementary Fig. 7C and F), which has been confirmed in AOA-dominated systems or pure cultures in previous studies [20, 62]. The proportion of hybrid N_2_O production was robust across water depths and stations (Supplementary Fig. 12), but showed variations across ocean regions, such as the Eastern Tropical North Pacific (95.6% ± 7.5% [63]), the South China Sea and the subtropical northwest Pacific (73.4% ± 14.0% [64]), and the Eastern Tropical South Pacific (70%–85% [65]). The proportions of hybrid N_2_O appear to be insensitive to the different nitrogen pools (NH_4_^+^, NO_2_^−^) and O_2_, which could indicate that the hybrid N_2_O formation does not involve external pools, or that the external pools are never constrained [63], since the turnover of the dissolved inorganic nitrogen pools is rapid turnover through microbial ammonium regeneration even in oligotrophic oceans [66]. In addition, pH decrease not only increased N_2_O production during the ammonia oxidation process but also significantly enhanced hybrid N_2_O production (slope = −0.253, *R*^2^ = 0.27, *P* < .001). In contrast, pH decrease enhanced the formation of ^46^N_2_O during urea oxidation (slope = +0.197, *R*^2^ = 0.35, *P* < .001) but reduced N_2_O yield (Supplementary Fig. 10). We hypothesize that pH changes caused differing responses in ammonia oxidation and urea oxidation rates, leading to reassignment of intracellular nitrogen intermediates [25, 55, 67]. NH_2_OH (pKa = 5.96) becomes more protonated and is more readily oxidized to HNO. Under acidic conditions, HNO undergoes non-enzymatic dimerization to N_2_O [19]. In contrast, the pH decrease enhances the proportion of HNO_2_ in NO_2_^−^, promoting NO production and contributing to the production of the hybrid N_2_O [68]. These processes provide an explanation for the observed N_2_O yield and pH-dependent isotopic shifts.

### Metagenomic evidence supports potential archaeal urea oxidation and nitrous oxide production

**Figure 4:**
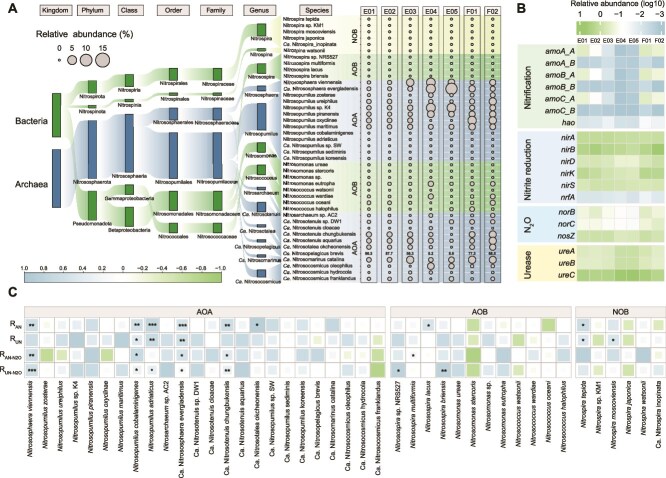
Linking nitrifying genes and microorganisms to the nitrification process. (A) Composition and relative abundance of nitrifying microorganisms based on metagenomic reads annotation. (B) The relative abundance of microbial functional traits in different stations. Only representative gene families related with nitrification, nitrite reduction, N_2_O cycle and urease were annotated. (C) Correlation analysis of the relative abundance of nitrifying microorganisms with the nitrification and the N_2_O production rates. Asterisks indicate the statistical significance (^***^P < .001; ^**^P < .01; and ^*^P < .05).

From metagenomes we recovered 3.55 × 10^6^ nitrogen-cycle gene reads (3.83–7.32 × 10^5^ per station, Supplemental Table S4). LEfSe showed significant regional differences (Fig. 4, Supplementary Fig. S13; Supplemental Table S5). Urease (*ureABC*) was enriched in the Philippine Basin than equatorial waters (LEfSe, *P* < .01), implicating active urea utilize in oligotrophic waters [9]. A total of 42 nitrifying microorganisms were identified, AOA were the dominant nitrifying microorganisms (relative abundance >78%) in WTP Ocean (Fig. 4B and Supplemental Fig. S14), as has been reported in previous studies [69]. Across stations, AOA abundance correlated positively with NH_4_^+^-driven nitrification and N_2_O production (e.g. *Nitrosopumilus* spp.), and several AOB lineages (e.g. *Nitrosomonas* spp./*Nitrosococcus* spp.) also showed positive associations, NOB displayed no consistent link to N_2_O (Fig. 4C and Supplemental Table S6). Comparative genomes analysis indicated >58% of AOA/AOB species contained genes encoding both ammonia monooxygenase and urease (Supplementary Fig. S14), suggesting widespread potential for urea utilize and oxidation in study waters.

**Figure 5:**
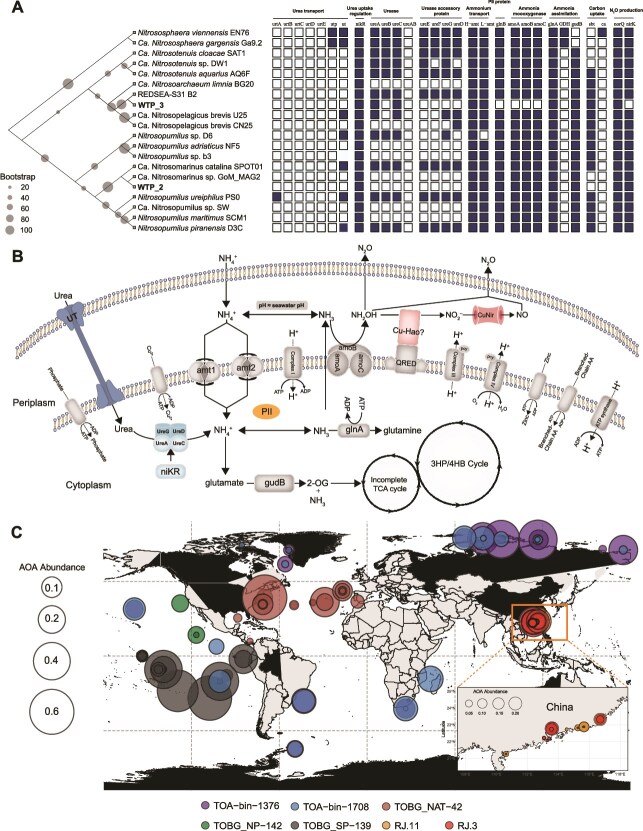
Genomic metabolic potential of recovered AOA MAGs and globally widespread urea-type AOA. (A) *amoA*-based phylogeny of AOA MAGs recovered in this study and the presence/absence of ammonia/urea uptake and utilization genes. Confidence values are based on 1000 bootstrap replications. Blue and white colors represent the presence and absence of the gene, respectively. (B) Schematic pathway reconstruction of the two metagenome-assembled genomes based on multiple annotations. Gray shapes indicate genes shared by NH_3_-type AOA and urea-type AOA, and blue shapes in dashed boxes indicate genes unique to urea-type AOA; urt: ATP-dependent urea ABC transporter; ut: urea transporter; *nikR*: nickel-responsive transcriptional regulator; *ureABC*: urease; *ureDEFG*: urease accessory protein; amt: Amt-type ammonium transporter; *glnB*: nitrogen regulatory PII protein; *glnA*: glutamine synthetase; GDH: glutamate dehydrogenase; *gudB*: glutamate dehydrogenase; sbt: bicarbonate transporter; ca: carbonic anhydrase. (C) Distribution of urea-type AOA in the world’s oceans, the size of the circle is proportional to relative abundance of each MAGs. Colored dots represent different reconstructed MAGs from Tara Oceans Expedition, Tara Oceans Polar Circle expedition and subtropical estuaries in South China.

We reconstructed 95 high-quality (completeness >90%, contamination <5%) and 275 medium-quality (completeness >50%, contamination <10%) metagenomic assembled genome (MAG) from extensive metagenomic reads. We recovered four ammonia-oxidizing archaeal MAGs (WTP_1, WTP_2, WTP_3, and WTP_4) from the metagenomes (Supplementary Table S7). Based on the archaeal 122 single-copy marker genes, we reconstructed a phylogeny that includes our MAGs (WTP_2 and WTP_3) and representative AOA genomes (Fig. 5A and Supplementary Fig. S15). WTP_2 affiliates with Ca. Nitrosomarinus-like AOA lineages, whereas WTP_3 clusters within Ca. Nitrosopelagicus-like close to REDSEA-S31_B2 (WCA clade) (Supplementary Table S7). WTP_3 lacks a recovered *amo* cluster, but it harbors *ureA*/*ureC* and regulatory elements (Fig. 5A and B). Considering potential assembly errors and the acquisition/loss dynamics of urea utilization genes [70], we do not infer the loss of ammonia oxidation capability in WTP_3 nor transfer functional annotations from the reference genome to our local population. Based on combined evidence from isotopic incubation and qPCR results, we have conclusive evidence indicating the widespread presence of ammonia-oxidizing microorganisms in the Western Pacific Ocean that utilize urea for the ammonia-oxidizing process and produce N_2_O.

To explore the global distribution of urea-type AOA (containing both archaeal *amoA* and *ureC* genes), we searched for AOA containing both archaeal *amoA* and *ureC* genes by comparison among 3661 microbial metagenome-assembled genomes reconstructed from the Tara Oceans Expedition [71], Tara Oceans Polar Circle expedition [72] and subtropical estuaries in South China [73], and finally obtained seven urea-type AOA MAGs (Fig. 5C and Supplementary Table S6). Our results show that urea-type AOA is widely distributed in the global oceans, especially in tropical and subtropical oceans, and even part of urea-type AOA has relatively high relative abundance in polar oceans extending to high latitudes [13]. The current view is that urea acts as an alternative nitrogen source in ammonium-deficient seawater [41]. However, we observed substantial urea-type AOA MAG abundances in eutrophic South China estuarine surface waters, and direct urea use by AOA has also been reported for the Gulf of Mexico [9]. Our data indicate that urea-type AOA are globally widespread, but the relative contributions of urea-driven nitrification and N_2_O production show covariations with the ratio of ambient urea to NH_4_^+^ [51]. Therefore, we recommend that future *in situ* and modeling studies explicitly evaluate the contribution of urea oxidation in natural environments to nitrification and N_2_O production, in order to better constrain its role in marine nitrification and N_2_O formation scenarios.

### Effects of microbial diversity and community structure to ocean acidification

A total of 66 nitrifying microbial ASVs were identified, of which 53 were annotated as AOA, 13 were annotated as NOB, and no AOB were detected. Our results showed that AOA was largely Ca. Nitrosopelagicus sp., and NOB was largely *Nitrospina* spp., and they are considered to be the dominant species of AOA and NOB in the oceans [5]. Experimental acidification did not lead to significant changes in the α-diversity of the nitrifying microbial community (Shannon: t = 2.43, *P* = .05; Observer ASVs: t = 1.11, *P* = .30) (Supplementary Fig. 16A and B), and NMDS analyses also indicated that the acidification treatments did not significantly alter the community composition of nitrifying microbes (PERMANOVA: F = 0.799, *P* = .491) (Supplementary Fig. 16D), which may be attributed to the adaptation of AOA to the environment [74, 75]. Random Forest based ranking of the relative contribution of ASVs showed that ASV_6 was the most important species in terms of relative importance to the whole community, with acidification treatments leading to the ASV_6 relative abundance increase from 61.7% to 77.4% (Supplementary Fig. S13C). Microbial co-occurrence networks were constructed based on SparCC correlations between ASVs to examine the effect of OA on microbial interactions (Supplementary Fig. S14). We found that the node numbers, edge numbers, and node degrees of the microbial networks increased in OA treated seawater compared to the control treatment (Supplementary Fig. S14), as did the proportion of positively correlated edges (52.6% to 53.9%), and that the increase in these features indicated that acidification leading to increased microbial network complexity and stronger interactions among microbes. The AOA-associated edge of the microbial network increased from 4.93% to 6.69% under acidification treatment, indicating that OA leads to more frequent interactions of AOA with other microbes [75].

### Mechanisms of nitrification and nitrous oxide production driven by microbial substrate preference

**Figure 6:**
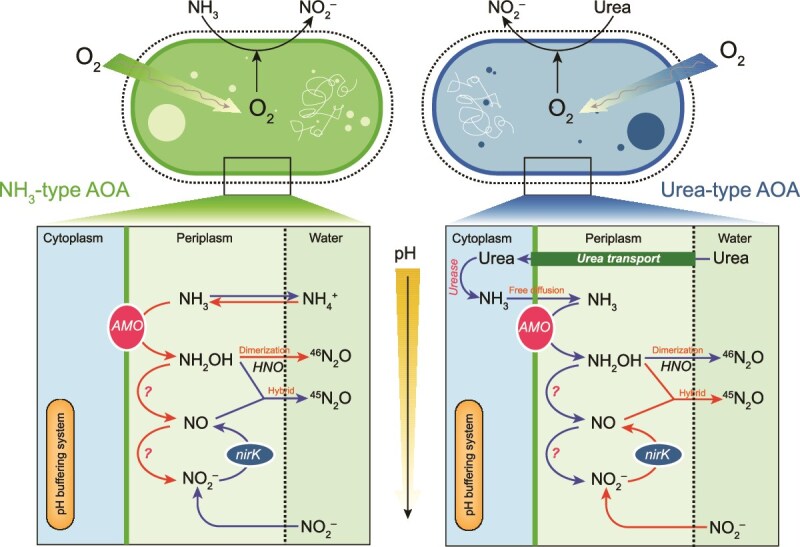
Conceptual scheme of the proposed nitrification and N_2_O production pathways for NH_3_-type AOA and urea-type AOA based on the different substrate sensitivities to pH. Subcellular compartments are shown (cytoplasm, periplasm, external seawater) together with the inferred localization of key functions: AMO on the inner membrane, urease in the cytoplasm, UT across the membrane, and Cu-nitrite reductase (*nirK*) in the periplasm. The vertical yellow arrow denotes a shift toward lower pH (acidification treatment). Red arrows mark steps expected to increase under lower pH (e.g. non-enzymatic dimerization of HNO to ^46^N_2_O, enhanced relative contribution of urease-supplied intracellular NH_3_), whereas blue arrows mark steps expected to decrease (e.g. external NH_3_ availability and free diffusion as the NH_3_/NH_4_^+^ equilibrium shifts). Hybrid ^45^N_2_O denotes coupling of ^15^NH_4_^+^-derived intermediates with an unlabeled N source (e.g. NO/NO_2_^−^). Question marks indicate unknown or debated steps. This schematic integrates our incubation and genome evidence and highlights trait differences between NH_3_- and urea-type AOA rather than asserting a single universal pathway.

Urea oxidation has been observed in various marine environments, via direct oxidation by ammonia-oxidizers and/or indirect pathway [4, 9]. In our experiments, the differential pH responses of ammonium- and urea-driven nitrification further support a primary direct pathway for urea, with only a minor indirect contribution. Our acidification experiments showed different responses of ammonium- and urea-driven nitrification, suggesting pH-modulated shifts in the intracellular precursor compounds for N_2_O. In AOA, NH_2_OH and NO are key intermediates [20, 21]. Because tracer enrichment exceeded 80% (^15^NH_4_^+^ and ^15^N-Urea), intracellular production largely yields ^15^NH_2_OH, so the isotopic composition of N_2_O is strongly governed by the intracellular NO/NO_2_^−^ pool. Given no confirmed hao homolog in AOA, NO likely derives from *CuNir*-mediated NO_2_^−^ reduction [76], making the isotopic signature of the intracellular NO_2_^−^ pool is critical for the isotopic composition of N_2_O. Ammonia oxidation rate can regulate the proportion of extracellular NO_2_^−^ that enters the intracellular NO_2_^−^ pool, and that higher ammonia oxidation rates may lead to overloading of the intracellular NO_2_^−^ pool, thereby inhibiting the entry of extracellular NO_2_^−^ into the cell [77]. With pH decreasing, ammonium-driven nitrification declines, which leads to a decrease in ^15^NO_2_^−^ entering the intracellular NO_2_^−^ pool, where the putative *CuNir* reduces NO_2_^−^ to NO, and the coupling of ^15^NH_2_OH to NO (whether enzymatically or non-enzymatic) would lead to the production of a large proportion of ^45^N_2_O (Fig. 6). ^15^NH_3_ arises intracellularly via urease, cytoplasmic pH homeostasis and broad AOA pH tolerance allow urea oxidation to persist or increase as external pH decline [10], consistent with *amoA* and *ureC* copies increases (Supplementary Fig. 8). In contrast, the urea-driven increase in ^46^N_2_O indicated that the large amount of “endogenous” ^15^NH_3_ produced by urea hydrolysis promotes the intracellular accumulation of ^15^NH_2_OH and ^15^NO_2_^−^ (Fig. 6). Limited leakage of endogenous ^15^NH_3_ to bulk water cannot be excluded but would be diluted and constrained by speciation. Recent work shows NH_2_OH oxidized to HNO (via copper enzymes) with non-enzymatic HNO dimerization to N_2_O is pH-sensitive and accelerated at low pH [19]. If this pathway is also active in natural seawater AOA communities, it provides an alternative explanation for our results. This dual microbial-abiotic mechanism integrates well with our genomic findings. Together with the widespread occurrence of *amoA*/*ureC*-bearing AOA in metagenomes, these results indicate that substrate and intermediate chemistry jointly contribute to N_2_O production and nitrogen pool balance under acidification, with urea-type AOA potentially gaining importance where conditions favor urea oxidation.

Our study provides new insights into marine nitrification by elucidating the processes and mechanisms of ammonium and urea-driven nitrification and N_2_O production in the euphotic zone of tropical western Pacific. With depth-integrated urea-driven nitrification rates contributing 14.2%–40.7% of total ammonium and urea nitrification rates, and depth-integrated urea-driven N_2_O production accounting for 20.6%–38.8% of total N_2_O production in nitrification. Our results confirm that urea-driven nitrification is critical for the balance of the nitrogen cycle in the oligotrophic ocean. In particular, the different responses of ammonium and urea-driven nitrification rates to pH decreases suggest that ammonia oxidation rates declines could be partially offset by increased urea oxidation rates under future OA scenarios. The widespread distribution of urea-type AOA in the global ocean indicates that urea-driven nitrification and N_2_O production may occur widely in the global ocean, and thus requires a revisiting of nitrification fluxes, as well as the associated dark carbon fixation, N_2_O production, and DO consumption within the ocean. To improve projections of total nitrification and marine N_2_O emissions under OA, Earth system N-cycle models should consider explicitly parameterizing urea oxidation and ammonium oxidation, with pH-sensitive yields and environment-dependent substrate partitioning, where supported by observations.

## Supplementary Material

Clean_SI_wraf281

Supplementary_Table_S1-S9_wraf281

## Data Availability

All sequencing products associated with this project can be found under National Center for Biotechnology Information (NCBI) BioProject ID PRJNA1248173. The AOAs reference genomes used for comparative genomic analysis were downloaded from the NCBI Refseq database.

## Code availability

The present study did not generate codes, and the mentioned tools used for the data analysis were applied with default parameters unless specified otherwise.
