# Supplementary material for: Urea-driven nitrification contributes to N2O production in the oligotrophic euphotic ocean
Source: ISME J. 2025 Dec 18;19(1):wraf281. doi: 10.1093/ismejo/wraf281 (PMC12774514; doi:10.1093/ismejo/wraf281)
Supplement: Clean_SI_wraf281 [file clean_si_wraf281.docx]

Supplementary Materials for

**Urea-driven nitrification contributes to N_2_O production in the oligotrophic euphotic ocean**

Running title: Urea-driven nitrification and N_2_O

Ting Gu^1,2^, Zhuo Chen^1,2^, David A. Hutchins^3^, Jun Sun^1,2*^

^1^Research Centre for Indian Ocean Ecosystem, Tianjin University of Science and Technology, Tianjin 300457, China

^2^State Key Laboratory of Geomicrobiology and Environmental Changes, China University of Geosciences (Wuhan), Wuhan, Hubei 430074, PR China

^3^Department of Biological Sciences, University of Southern California, Los Angeles, CA 90089, USA

*Corresponding author. Jun Sun, Research Centre for Indian Ocean Ecosystem, Tianjin University of Science and Technology, No. 9, 13th Avenue, Tianjin Economic-Technological Development Area, Tianjin, 300457, P. R. China. Email: phytoplankton@163.com

**This file includes:**

Supplementary Figs. 1 to 17

Supplementary methods

Supplementary Table S1 to S9

References


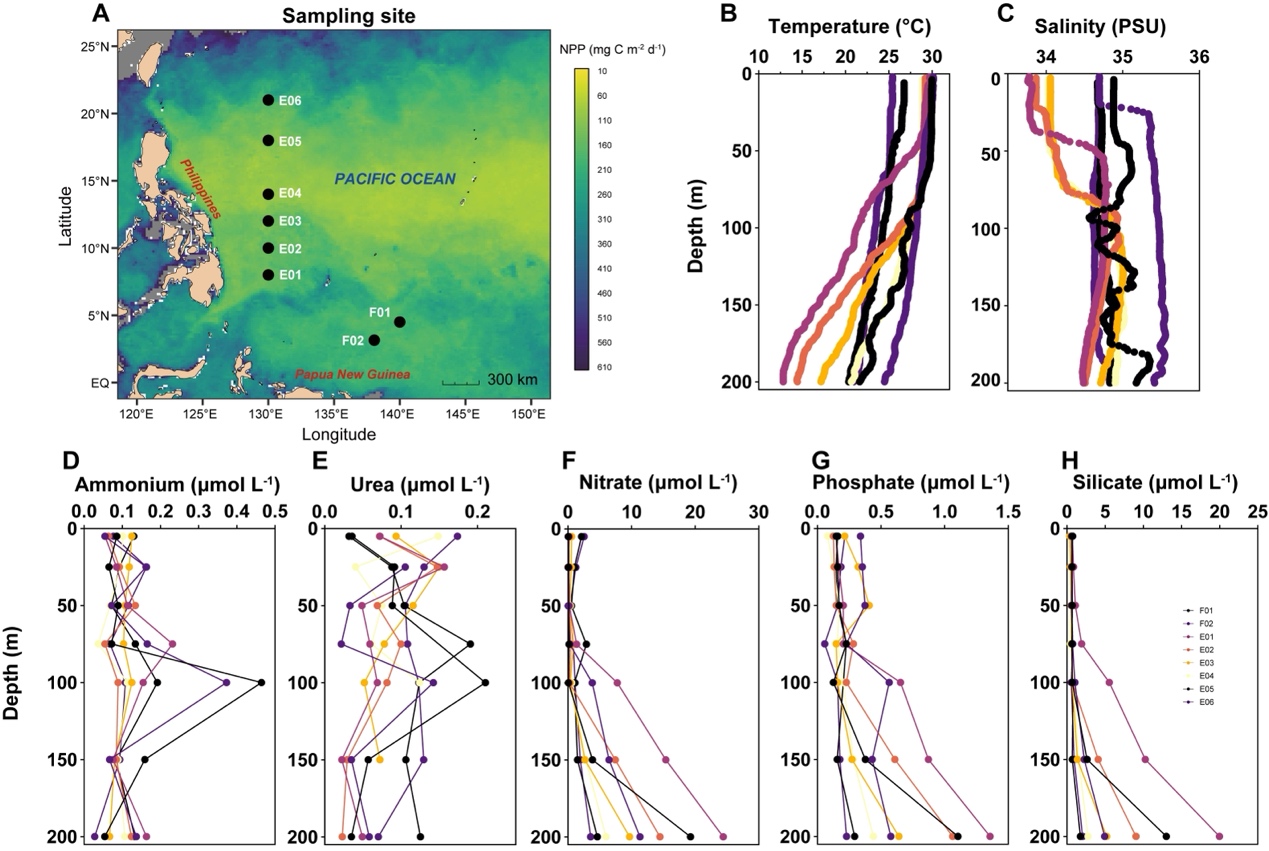


**Supplementary Fig. 1. Sampling stations and water column physicochemical parameters in the western tropical Pacific Ocean**. **A**, Sampling stations are marked with black dots and map background color-filled with 2023 average annual net primary productivity (NPP, mg C m^-2^ d^-1^), data from Ocean Productivity Standard VGPM NPP Products (http://orca. science.oregonstate.edu/npp_products.php). **B-C**, Temperature and salinity data at study stations. **D-H**, Ammonium, urea, nitrate, phosphate, and silicate concentrations at study stations. Colors indicate different stations.


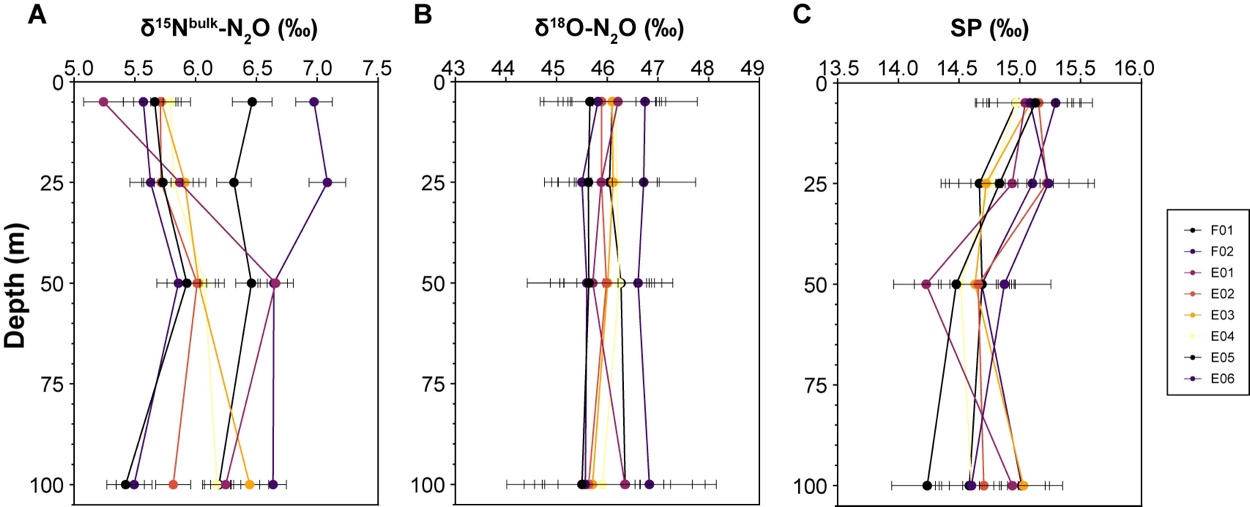


**Supplementary Fig. 2. Depth profile of N_2_O isotopomers.** **A**, δ^15^N^bulk^-N_2_O (‰). **B**, δ^18^O-N_2_O (‰). **C**, Site preference (‰). Colors indicate different stations. Symbols show values at 5, 25, 50, and 100 m. Horizontal whiskers indicate ±SD.


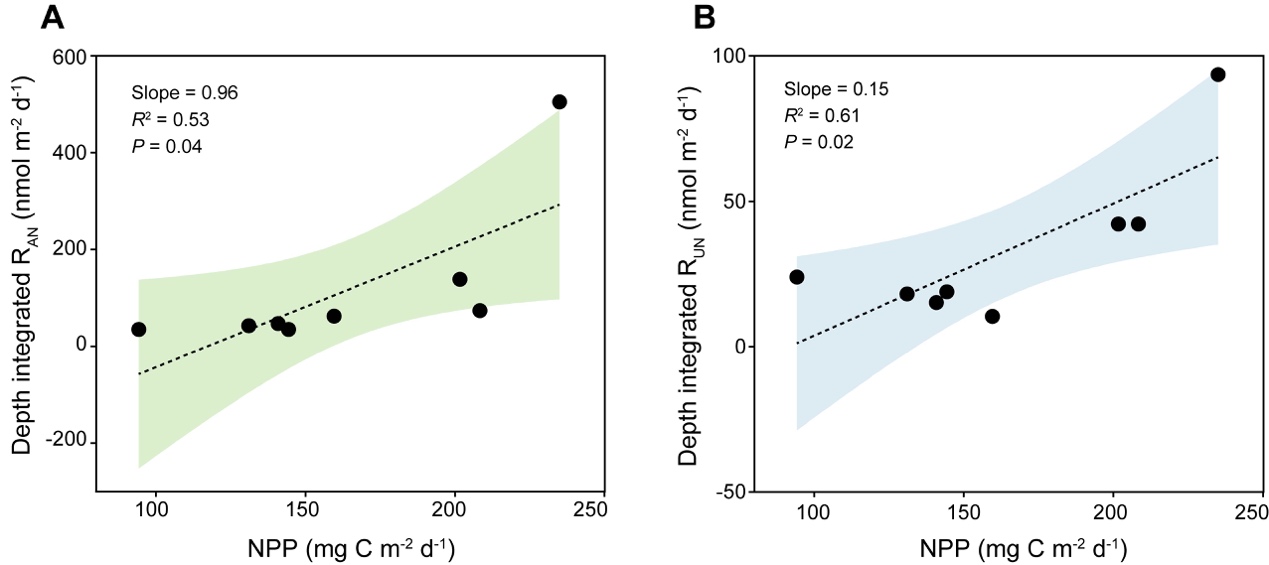


**Supplementary Fig. 3. Correlation between the depth-integrated nitrification rates and the net primary productivity**. **A-B**, Depth-integrated ammonium/urea-driven nitrification rate are positively correlated with net primary productivity. *R*^2^ represents the goodness of fit, *P* value < 0.05 indicates significant correlation.


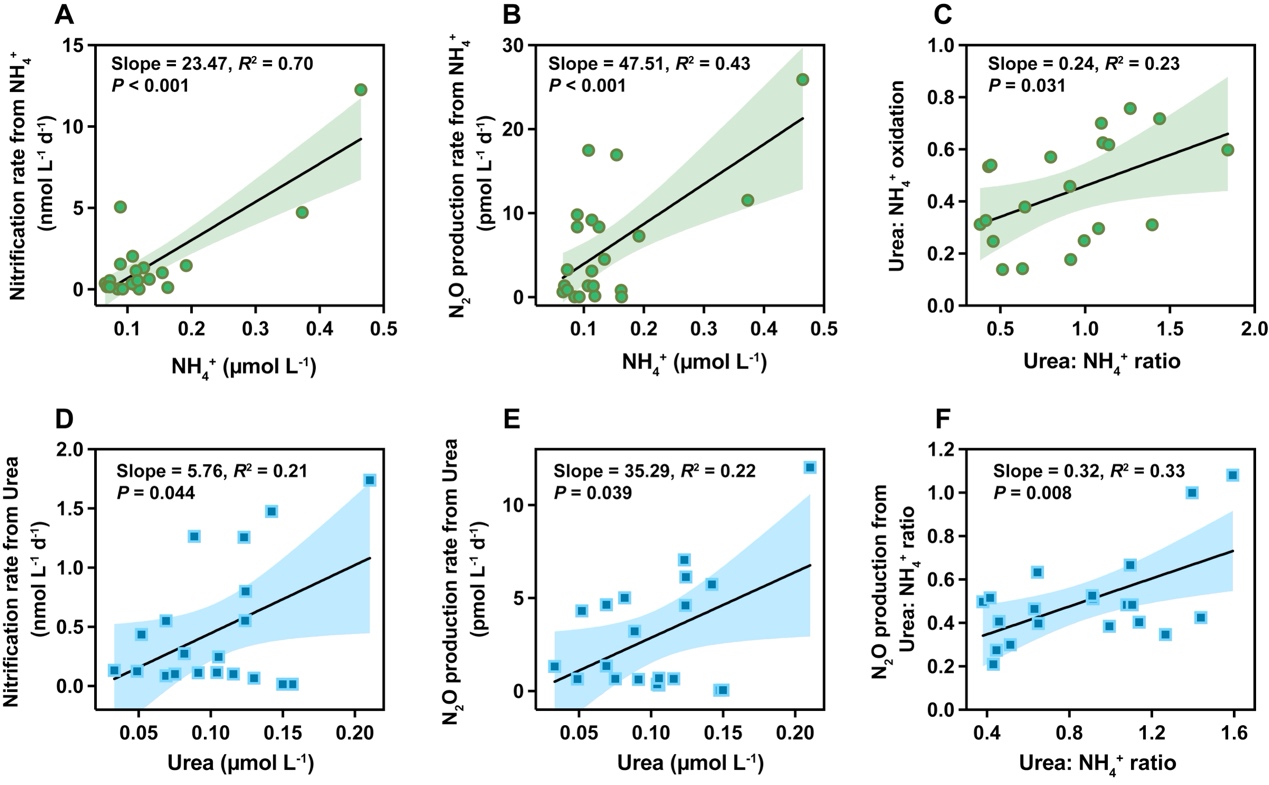


**Supplementary Fig. 4. Substrate concentrations and process rates and cross-substrate ratios.** **A**, Nitrification rate from NH_4_^+^ (nmol L^-1^ d^-1^) and NH_4_^+^ (µmol L^-1^), **B**, N_2_O production rate from NH_4_^+^ (pmol L^-1^ d^-1^) vs NH_4_^+^, **C**, Ratio of urea- to NH_4_^+^-driven oxidation with the urea:NH_4_^+^ concentration ratio, **D**, Nitrification rate from urea (nmol L^-1^ d^-1^) and urea (µmol L^-1^), **E**, N_2_O production rate from urea (pmol L^-1^ d^-1^) and urea, **F**, Ratio of urea- to NH_4_^+^-derived N_2_O production with the urea:NH_4_^+^ concentration ratio. Solid lines are ordinary least squares fits, shaded bands show 95% confidence intervals. Slope, *R*^2^ and *P* value are reported. Only data above the method detection limit are plotted.


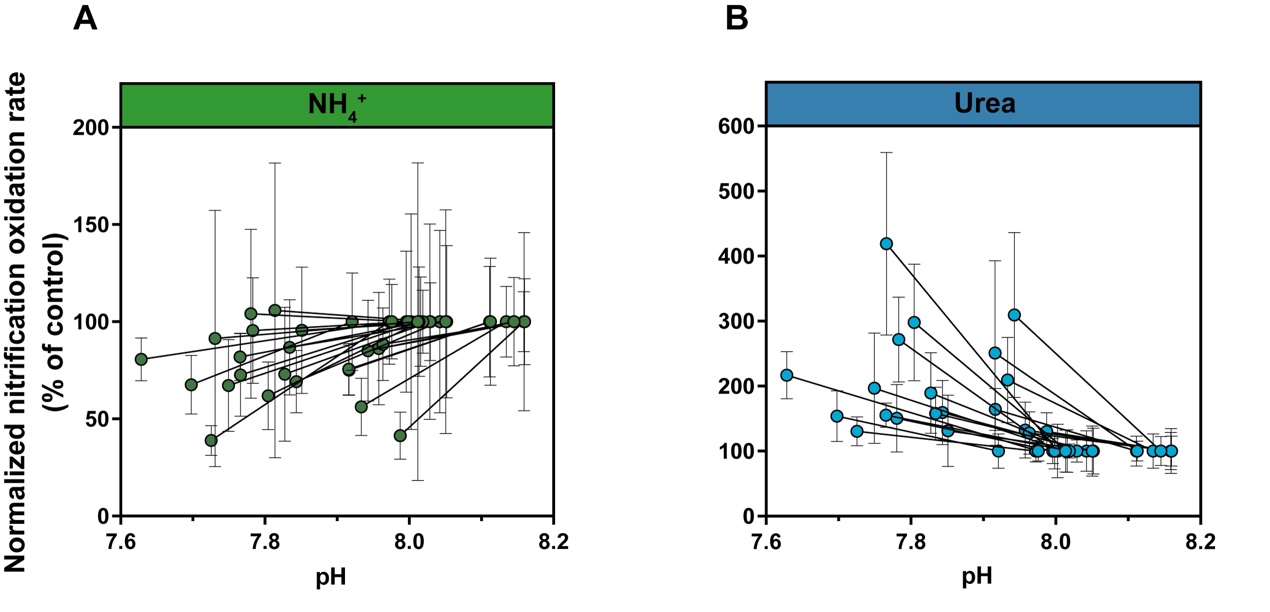


**Supplementary Fig. 5. Correspondence between nitrification rates and pH for all experiments. A,** ammonium-driven nitrification rate, **B**, urea-driven nitrification rate. Nitrification rates were renormalized to the control measured in the experiment.


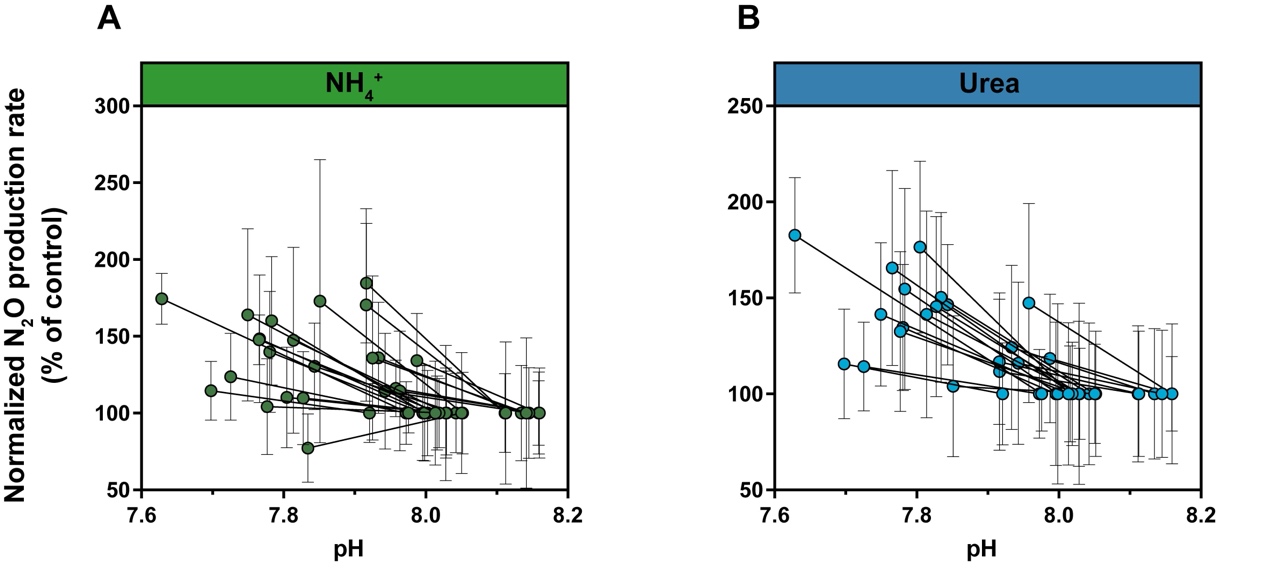


**Supplementary Fig. 6. Correspondence between N_2_O production rates and pH for all experiments. A,** ammonium-driven N_2_O production rates, **B**, urea-driven N_2_O production rates. N_2_O production rates were renormalized to the control measured in the experiment.


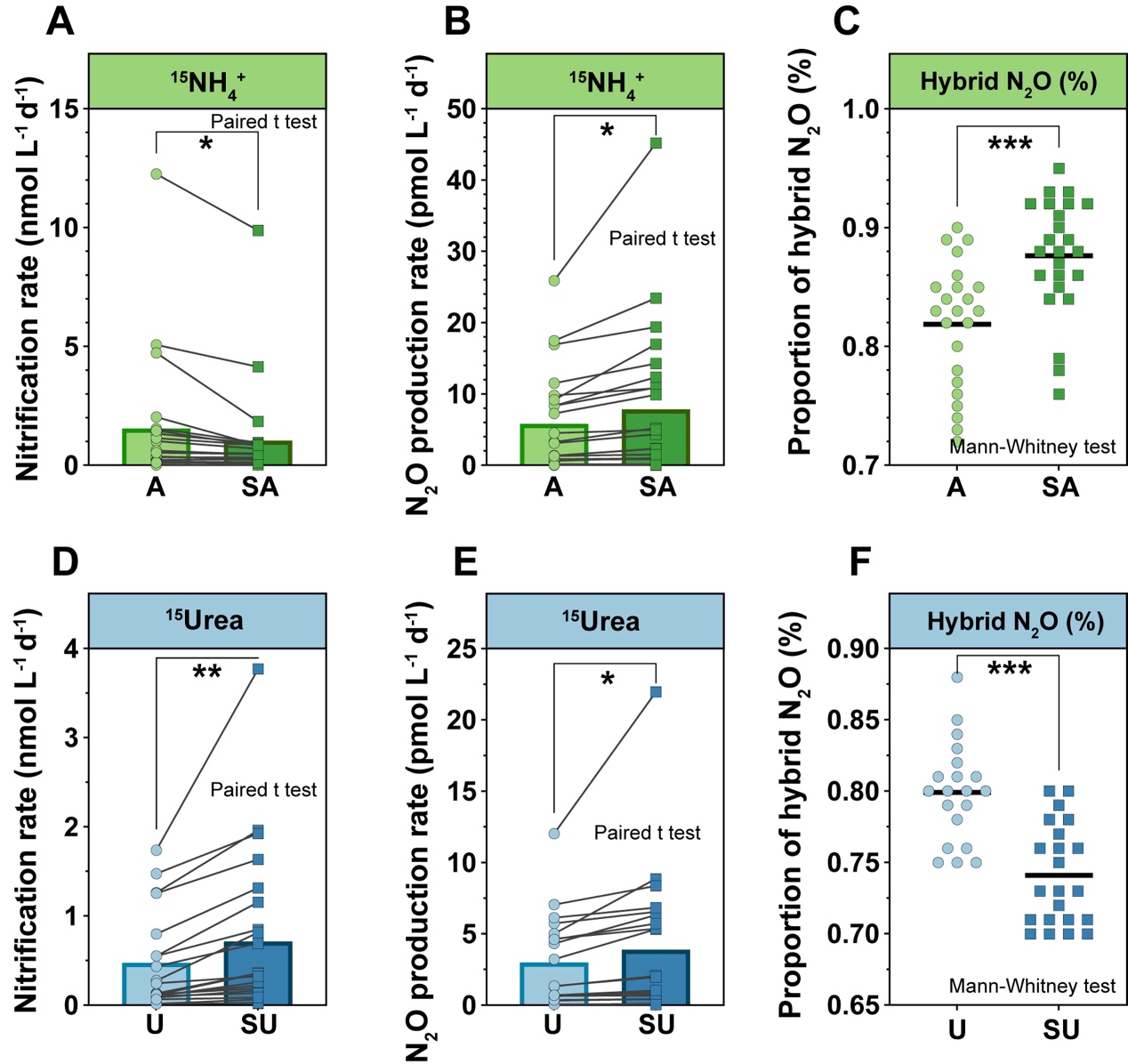


**Supplementary Fig. 7. Response of nitrification rate, nitrous oxide production rate and proportion of hybrid N_2_O to ocean acidification.** **A**, Ammonium-driven nitrification rates. **B**, Ammonium-driven N_2_O production rates. **C**, ammonium-driven hybrid N_2_O proportion. **D**, Urea-driven nitrification rates. **E**, Urea-driven N_2_O production rates. **F**, urea-driven hybrid N_2_O proportion. In **C** and **F**, black dots represent control treatments, black squares represent acidification treatments and color fills represent 95% confidence intervals. Statistical of difference analyses were performed using paired samples t-tests, and Mann-Whitney test; * *P* < 0.05, ** *P* < 0.01, *** *P* < 0.001.


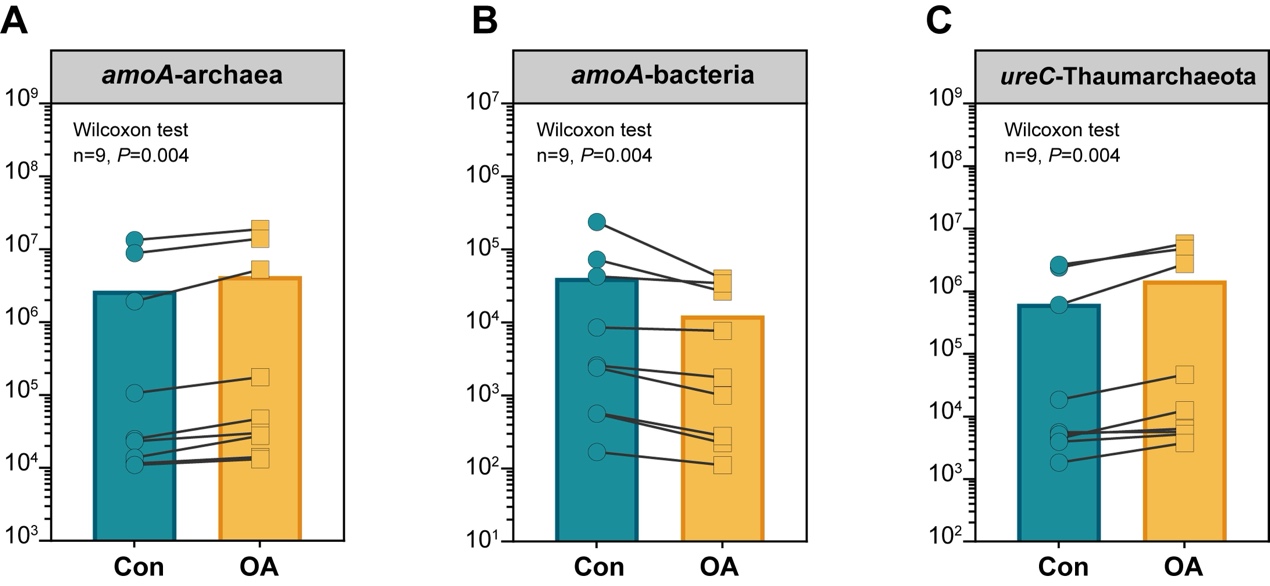


**Supplementary Fig. 8. Abundance of ammonia-oxidizing microorganisms based on qPCR technology**. **A**, Archaeal *amoA*, **B**, Bacteria *amoA*, **C**, Thaumarchaeota *ureC* in the ambient control and pH decrease treatments.


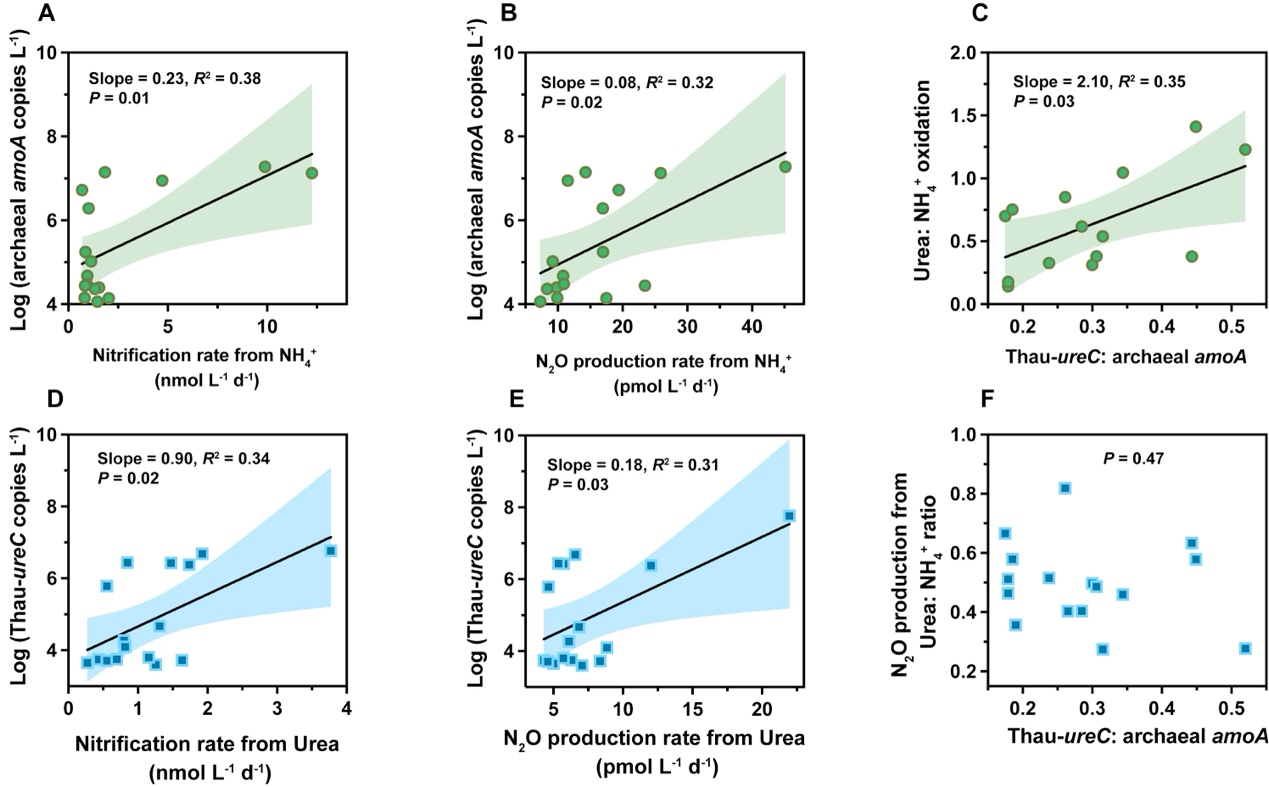


**Supplementary Fig. 9. Gene abundance link substrate-specific rates and N_2_O production.** **A–B**, Relationships between archaeal *amoA* gene abundance (log_10_ copies L^-1^) and (A) NH_4_^+^-driven nitrification rates (nmol L^-1^ d^-1^) and (B) NH_4_^+^-driven N_2_O production rates (pmol L^-1^ d^-1^). **C**, Ratio of urea:NH_4_^+^ oxidation versus the ratio Thau-*ureC* : archaeal *amoA*. **D–E**, Relationships between Thau-*ureC* gene abundance (log_10_ copies L^-1^) and (D) urea-driven nitrification rates (nmol L^-1^ d^-1^) and (E) urea-driven N_2_O production rates (pmol L^-1^ d^-1^). F, N_2_O production ratio (urea:NH_4_^+^) versus Thau-*ureC* : archaeal *amoA*. Only data above the method detection limit are plotted. Black lines are ordinary least squares fits; shaded bands denote 95% confidence intervals. Reported slope, *R*^2^, and *P* values are from the linear models.


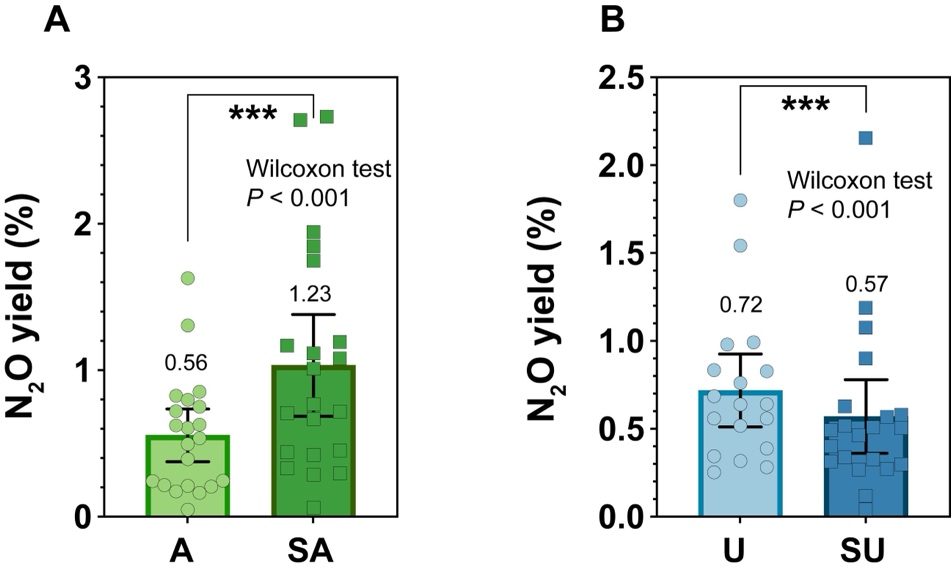


**Supplementary Fig. 10.** **Effects of acidification on N_2_O yield from nitrification.** **A**, NH_4_^+^-driven N_2_O yield (%) under ambient pH (A) and acidified (SA) conditions. **B**, Urea-driven N_2_O yield (%) under ambient pH (A) and acidified (SA) conditions. Bars show the mean ± SD, numbers above bars denote the mean. Differences between ambient and acidified treatments were tested with a Wilcoxon signed-rank test (*** *P* < 0.001).


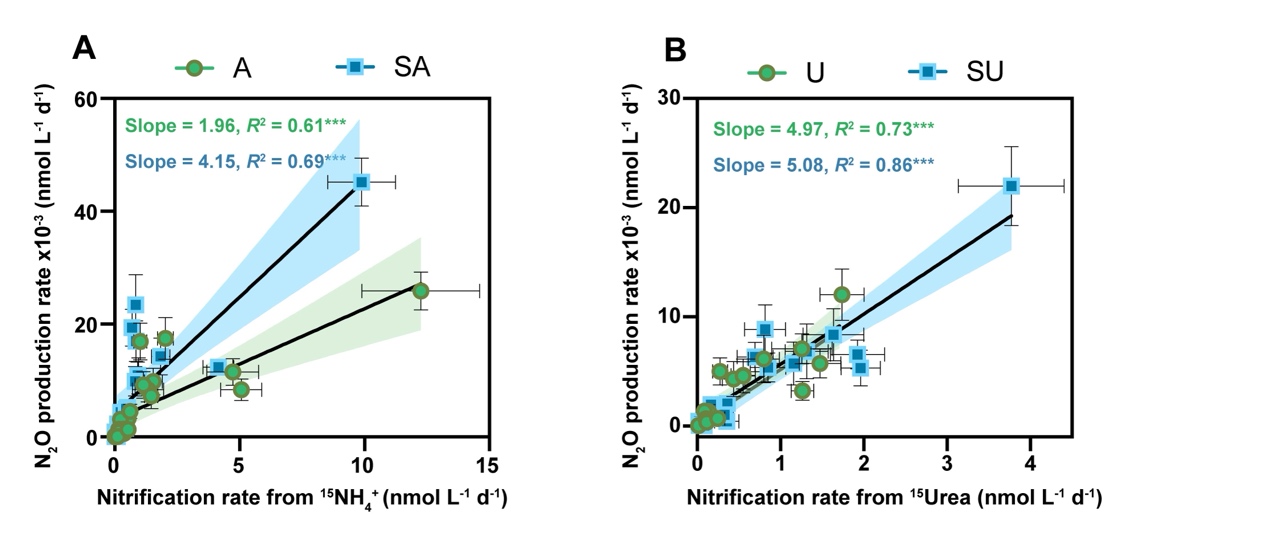


**Supplementary Fig. 11. Coupling between nitrification and N_2_O production under ambient and pH decline.** **A**, NH_4_^+^-driven rate under ambient (green circles) and acidification (blue squares). B, -driven rate under ambient (green circles) and acidification (blue squares). Only rates above detection limits are shown. Solid lines are ordinary least squares fits with 95% confidence bands (shaded). Reported slopes represent the apparent N_2_O yield, *R*^2^ indicates goodness of fit (*** *P* < 0.001).


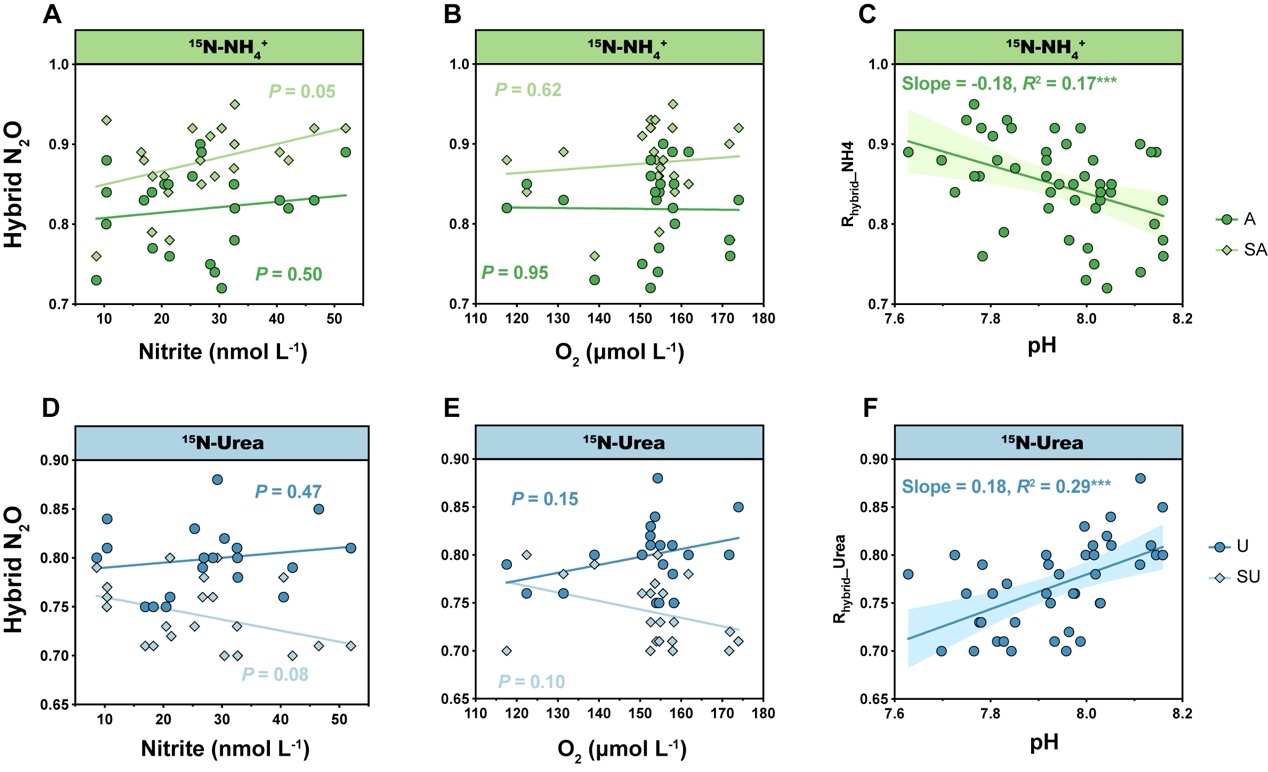


**Supplementary Fig. 12. Environmental controls on the hybrid N_2_O production fraction during ^15^N-labeled substrate incubations under control (Con) and acidification (OA) treatments. A-C**, relationships between hybrid N_2_O and environmental variables (nitrite, oxygen, and pH) under ^15^N-NH_4_^+^ additions. **D-F**, corresponding relationships under ^15^N-urea additions. Significant linear relationships are highlighted with regression lines and 95% confidence intervals (shaded areas). Acidification (OA) markedly decreased hybrid N_2_O under ^15^N-NH_4_^+^ (**C**) but increased it under ^15^N-urea (**F**). Circles denote control treatments, and diamonds represent acidification treatments. Slope, *R*^2^, and *P* values are presented for each regression, asterisks indicate statistical significance (****P* < 0.001).


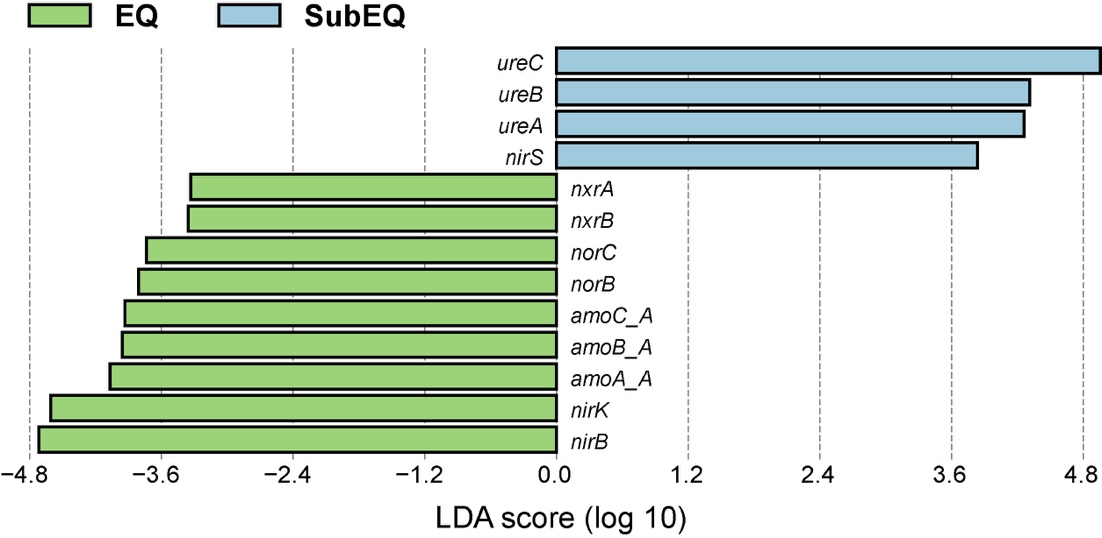


**Supplementary Fig. 13. LEfSe analysis of nitrogen cycling genes in equatorial and sub equatorial waters.**

**
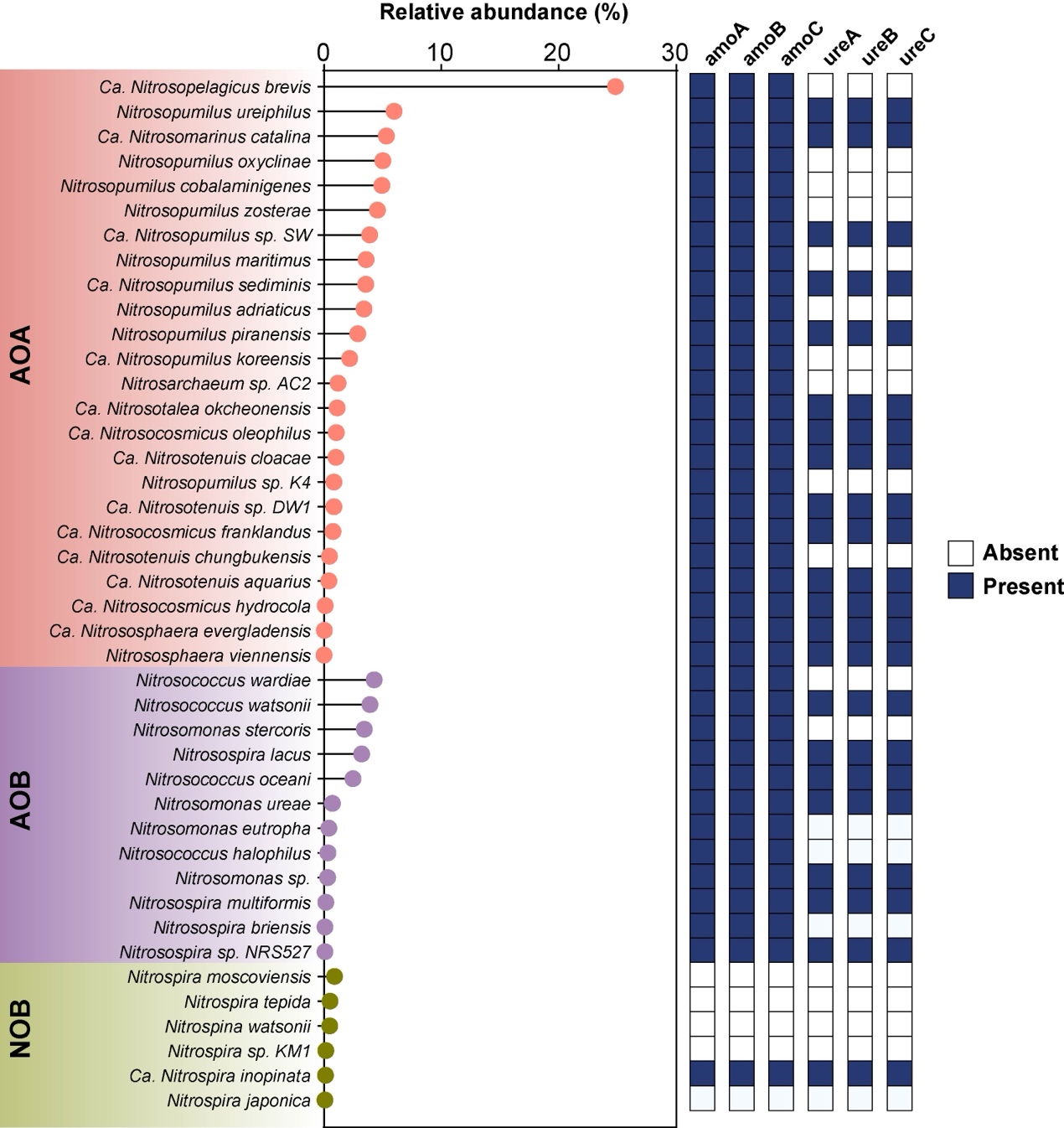
**

**Supplementary Fig. 14. Relative abundance of nitrifying microorganisms and gene annotation of representative species based on Kraken 2 annotation.**


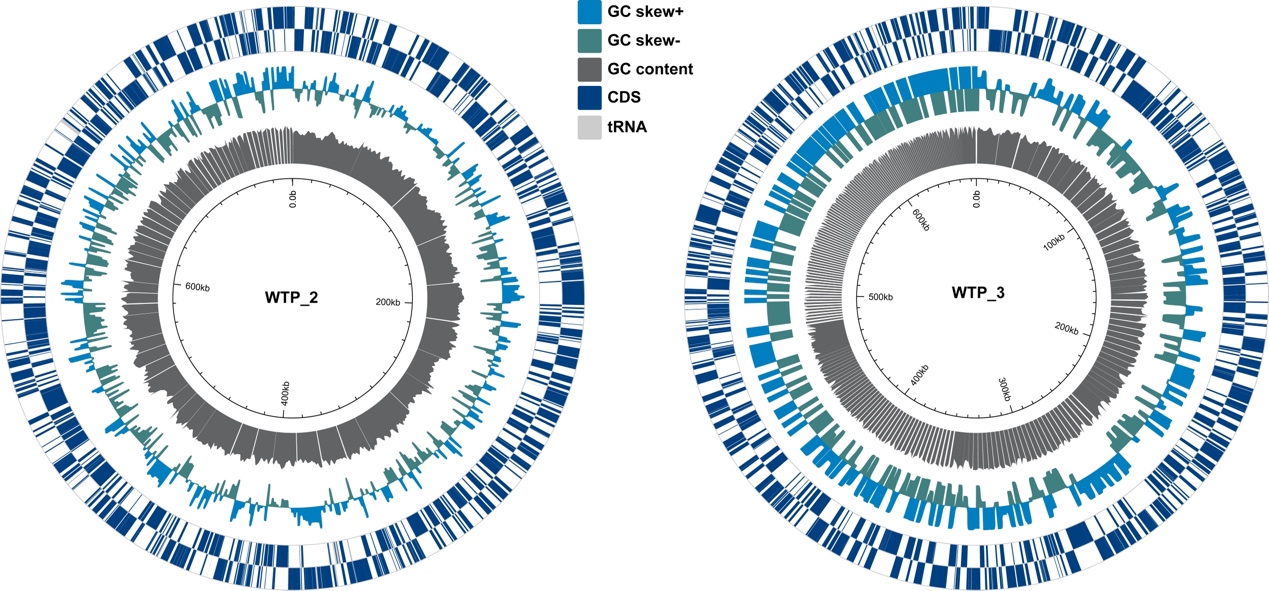


**Supplementary Fig. 15.** **Circular genome maps of AOA MAGs.** Circular maps showing the genomic architecture of two archaeal MAGs. GC content (grey histogram), GC skew, predicted coding sequences (CDS) on both strands (dark-blue bars), tRNA genes (light-grey ticks). Radial rulers indicate genome coordinates (kb).


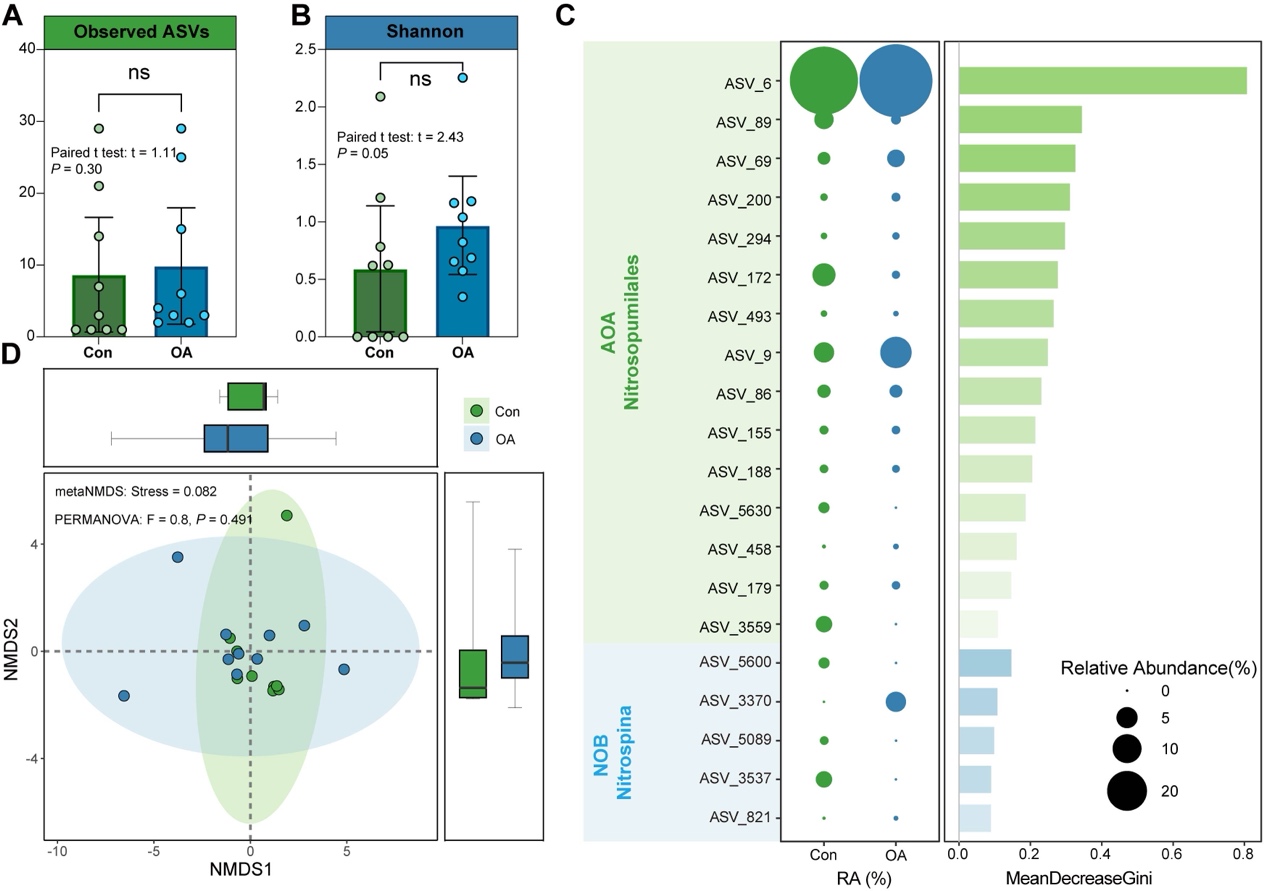


**Supplementary Fig. 16. Effect of ocean acidification on microbial diversity and community composition. A, B** Shannon and Chao1 indexes among control and acidification treatments. **C**, Relative importance ranking based on the random forest model. **D**, NMDS (Non-metric Multidimensional Scaling) analysis of community composition among control and acidification treatments. Stress value indicates the degree of difference between the interpoint distances in the downscaled results and the original distances. Statistical of difference analyses were performed using paired samples t-tests, with t values representing t statistics and n representing samples sizes; PERMANOVA (Permutational Multivariate Analysis of Variance) used to examine variability in species composition across treatments. *P* values < 0.05 indicate significant differences.


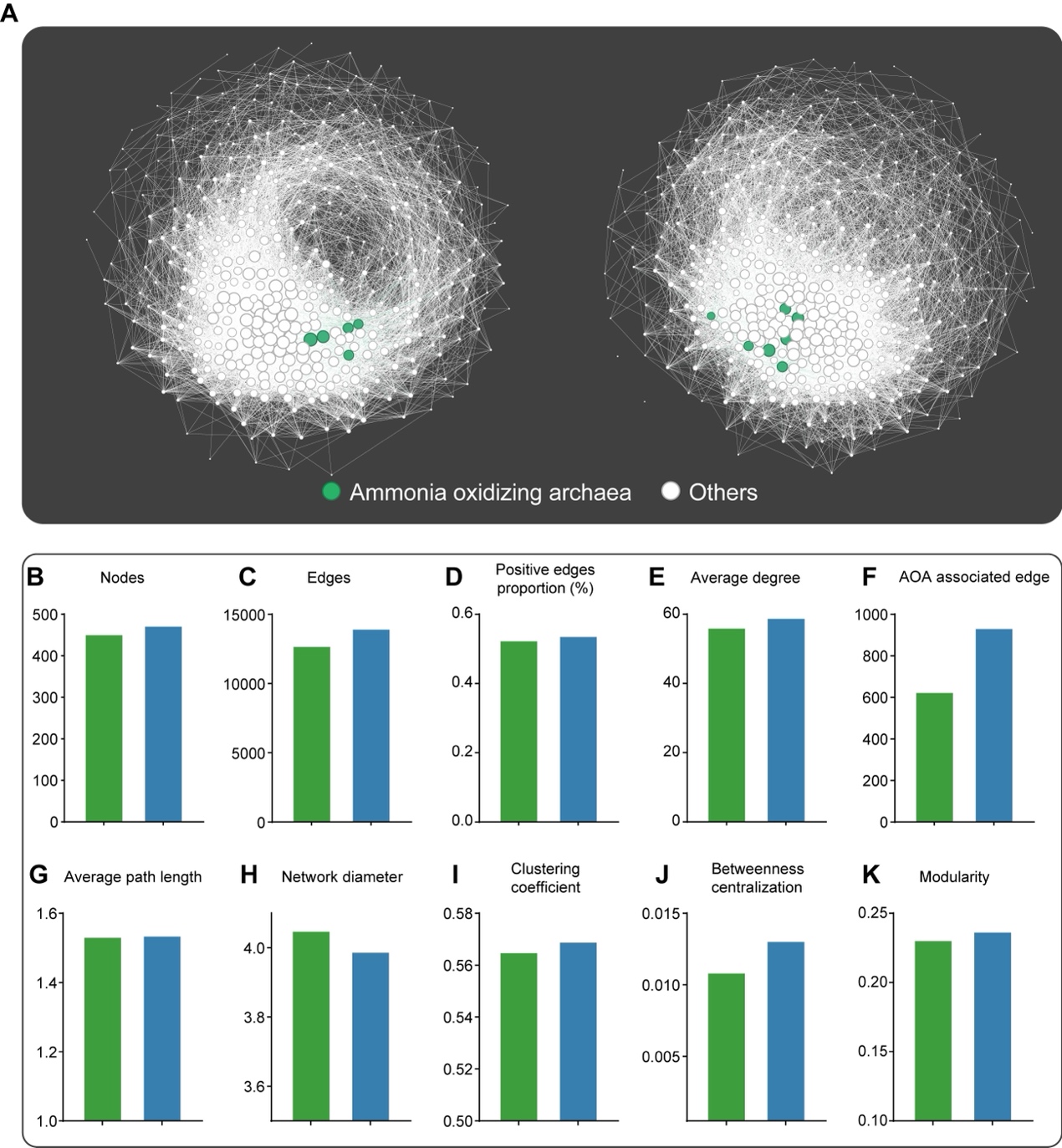


**Supplementary Fig. 17. Microbial network interaction analysis and topological parameters.** **A**, Co-occurrence network based on SparCC correlations, with AOA species labeled in green and the others in gray, and edges associated with AOA in green. **B-K**, Network topology parameters in control treatment and pH decrease treatment.

**Supplementary methods**

**Study area and field sampling**

This study was conducted on board the RV KEXUE during cruise NORC 2022-09 (February 14-April 15, 2023) in the tropical western Pacific. There was a total of eight sampling stations on this cruise, two of which were near the equator and the other six were in the Philippine Sea (Supplementary Fig. 1). Temperature, salinity, depth and dissolved oxygen data were obtained using a Seabird SBE 911 CTD sensor equipped with an oxygen sensor. Bulk seawater samples were collected using 24 10L Niskin bottles mounted on CTD rosette. The mixed layer depth was defined as the depth at which the difference of 0.8°C relative to the surface value was observed[1].

Samples for the determination of N_2_O concentration and isotopic abundance were collected using 120 ml clamped glass serum bottles (acid washed). Before sampling, the bottles were lubricated with seawater for three times, and the sampling tubes were inserted to the bottom of the bottles to allow water slowly to fill up the bottles and overflow more than twice of the bottle volumes. The sample tube was slowly removed and air bubbles were avoided throughout the procedure, 200 μL of saturated HgCl_2_ solution was quickly added to stop the microbial activity, and the samples were stored at 4℃ in dark. For nutrient samples filter 100 ml of seawater using a 0.2 μm syringe filter and store at -20°C. For chlorophyll *a* sample, 2 liters of seawater were slowly filtered through pre-combustion (450°C, 4 h) GF/F filters (Whatman, 25 mm diameter). After filtration, the filters were folded and wrapped in pre-combustion (450°C, 4 h) aluminum foil and stored at -20°C. Metagenomic samples were filtered through 20 L of seawater using a 0.2 μm filter membrane (Millipore Isopore) directly with a peristaltic pump, and the filter samples were preserved in liquid nitrogen.

**Nutrients and chlorophyll *a* measurements**

The concentrations of NH_4_^+^, NO_2_^−^ and NO_3_^−^ were determined using an AA3 AutoAnalyzer (SEAL Analytical) with a detection limit of 0.02 μmol L^-1^ for NH_4_^+^, 0.003 μmol L^-1^ for NO_2_^−^ and 0.02 μmol L^-1^ for NO_3_^−^, the precision was less than 3%. Based on the colorimetric reaction with diacetyl monoxime, the urea concentration was measured using capillary cell with a detection limit of 1 nmol N L^-1^[2], urea concentrations are reported as nitrogen-equivalent (urea-N) following the diacetyl-monoxime colorimetric method. For the determination of chlorophyll *a*, the standard solution was configured using chlorophyll *a* standards (99%, Sigma), and the filters were extracted using a 9:1 aqueous acetone solution for more than 12 h. After centrifugation at 4500 rpm, the supernatant was measured using a fluorescence photometer (Hitachi F-4700, Japan).

**Nitrogen cycling genes and microorganisms were annotated based on metagenomic reads**

Search for merged metagenomic sequences against the most complete database of nitrogen cycle gene families, NCycDB (https://github.com/qichao1984/NCyc), a manually curated functional gene database dedicated to the analysis of N-cycle pathways in metagenomes[3]. The entire NCycDB (68 gene families) was used for metagenomic analysis of nitrogen cycle communities. The nucleotide sequences of NCycDB were searched using the DIAMOND program (version 0.9.25) with blastx mode[4]. DIAMOND used parameters such as -k 1-e 0.0001. Functional profiles were obtained using the perl script provided in NCycDB. The total number of sequences per sample was normalized to 100, 000 ,000. To obtain taxonomic features of the microbial communities driven by the marine nitrogen cycle, sequences targeted by the nitrogen cycle gene family in NCycDB were extracted using the seqtk program (https://github.com/lh3/seqtk). The extracted sequences were then annotated by Kraken 2[5]. For annotation assignment, a local standard Kraken 2 database was created. Then, taxonomic maps of nitrogen cycle pathways were generated at different taxonomic levels.

**Assembly, binning, reassembly, and gene annotations**

To obtain genomic maps of ammonia oxidizing microorganisms and to understand their genomic metabolic potential. Genome assembly and binning was performed using the metaWRAP (version 1.3) pipeline[6]. Clipped reads were assembled using Megahit (version 1.2.9) to produce clean contigs. Then CONOCT (version 1.1.0)[7], MaxBin2 (version 2.2.7)[8], and MetaBAT (version 2.12.1)[9] were performed binning analysis of contigs over 1000 bp. The reconstructed bins were transferred to the bin collection within the bin_refinement module and modified with the Reassembly_bins module to obtain a metagenomic assembly genome (MAG). The quality of the obtained MAGs was checked using CheckM (version 1.2.2), and MAGs with completeness≤50% and contamination≥10% were removed. Taxonomic affiliation of MAGs was annotated by GTDB-Tk (version 2.3.2), and the 95% average nucleotide identity (ANI) threshold was used as the threshold.

In this study, we focused on MAG associated with ammonia oxidizing microorganisms. The multiple annotation method was used to obtain functional annotations for the interested MAGs. Specifically, Prodigal (version 2.6.3)[10] was used to predict protein-coding genes and initial functional annotations were obtained using Prokka (1.14.5). GhostKOALA (version 3.1) and eggnog-mapper (version 2.1.12) were used to search the NCBI Non-Redundant Protein Database via BLASTP to annotate the interested genes. Phylogenetic analyses based on the archaeal amoA gene were performed using iqtree2 (version 2.3.6)[11], and the phylogenetic tree was inferred using the Approximate Likelihood Ratio Test as well as 1,000 bootstraps and visualized using iTOL (version 6.0).

**Identification of urea-type AOA from global oceans**

For urea-type AOA searches, we used DIAMOND (version 0.9.25) in blastx mode to query NCycDB[3] for high-quality MAGs from the Tara Oceans Expedition[12], Tara Oceans Polar Circle expedition[13] and subtropical estuaries in South China[14] reconstructed 3661 MAGs. There are three ways to ensure the accuracy of urea-type AOA identification: (i) in running DIAMOND, strict parameters were set to identify amoA/B/C and *ureA/B/C* genes (e-value ≤ 1e-10, coverage ≥ 0.8, and homogeneity ≥ 0.9); (ii) the results based on NCycDB were compared with those based on KEGG, to ensure comparative accuracy of the comparison; (iii) only archaea containing both *amoA* and *ureC* genes were recognized as urea-type AOA. Methods for estimating the relative abundance of MAGs varied between samples, and the Tara Ocean expedition was based on metagenomic reads from secondary overlapping clusters for each sample using BEDTools (v 2. 17. 0; multicov default parameters) to estimate the length-normalized relative abundance of each MAGs[12]. The Tara Oceans Polar Circle expedition used BBTools (v.38.08) to compare read sequences from the samples to the reference genome (or draft genomes) to assess the abundance of genomes in each sample[13]. The estuarine metagenome samples were used to calculate the length-weighted average relative abundance of each MAG using the Quant_bins module in metaWRAP[14]. Given the differences in MAG relative abundance estimation methods, no horizontal comparative analyses were performed between samples.

**References**

1. Kara AB, Rochford PA, Hurlburt HE. An optimal definition for ocean mixed layer depth. *J Geophys Res-Oceans* 2000;**105**:16803–16821. https://doi.org/10.1029/2000JC900072

2. Chen L, Ma J, Huang Y, et al. Optimization of a colorimetric method to determine trace urea in seawater. *Limnol Oceanogr Meth* 2015;**13**:303–311. https://doi.org/10.1002/lom3.10026

3. Tu Q, Lin L, Cheng L, et al. NCycDB: a curated integrative database for fast and accurate metagenomic profiling of nitrogen cycling genes. *Bioinformatics* 2019;**35**:1040–1048. https://doi.org/10.1093/bioinformatics/bty741

4. Buchfink B, Xie C, Huson DH. Fast and sensitive protein alignment using DIAMOND. *Nat Methods* 2015;**12**:59–60. https://doi.org/10.1038/nmeth.3176

5. Wood DE, Lu J, Langmead B. Improved metagenomic analysis with Kraken 2. *Genome Biol* 2019;**20**:257. https://doi.org/10.1186/s13059-019-1891-0

6. Uritskiy GV, DiRuggiero J, Taylor J. MetaWRAP-a flexible pipeline for genome-resolved metagenomic data analysis. *Microbiome* 2018;**6**:158. https://doi.org/10.1186/s40168-018-0541-1

7. Alneberg J, Bjarnason BS, de Bruijn I, et al. Binning metagenomic contigs by coverage and composition. *Nat Methods* 2014;**11**:1144–1146. https://doi.org/10.1038/NMETH.3103

8. Wu Y-W, Simmons BA, Singer SW. MaxBin 2.0: an automated binning algorithm to recover genomes from multiple metagenomic datasets. *Bioinformatics* 2016;**32**:605–607. https://doi.org/10.1093/bioinformatics/btv638

9. Kang DD, Li F, Kirton E, et al. MetaBAT 2: an adaptive binning algorithm for robust and efficient genome reconstruction from metagenome assemblies. *PeerJ* 2019;**7**:e7359. https://doi.org/10.7717/peerj.7359

10. Hyatt D, Chen G-L, LoCascio PF, et al. Prodigal: prokaryotic gene recognition and translation initiation site identification. *BMC Bioinformatics* 2010;**11**:119. https://doi.org/10.1186/1471-2105-11-119

11. Minh BQ, Schmidt HA, Chernomor O, et al. IQ-TREE 2: New models and efficient methods for phylogenetic inference in the genomic era. *Mol Biol Evol* 2020;**37**:1530–1534. https://doi.org/10.1093/molbev/msaa015

12. Tully BJ, Graham ED, Heidelberg JF. The reconstruction of 2,631 draft metagenome-assembled genomes from the global oceans. *Sci Data* 2018;**5**:170203. https://doi.org/10.1038/sdata.2017.203

13. Royo-Llonch M, Sanchez P, Ruiz-Gonzalez C, et al. Compendium of 530 metagenome-assembled bacterial and archaeal genomes from the polar Arctic Ocean. *NAT MICROBIOL* 2021;**6**:1561–1574. https://doi.org/10.1038/s41564-021-00979-9

14. Zhou L, Huang S, Gong J, et al. 500 metagenome-assembled microbial genomes from 30 subtropical estuaries in South China. *Sci Data* 2022;**9**:310. https://doi.org/10.1038/s41597-022-01433-z

15. Frame CH, Casciotti KL. Biogeochemical controls and isotopic signatures of nitrous oxide production by a marine ammonia-oxidizing bacterium. *Biogeosciences* 2010;**7**:2695–2709. https://doi.org/10.5194/bg-7-2695-2010

16. Jung M-Y, Well R, Min D, et al. Isotopic signatures of N_2_O produced by ammonia-oxidizing archaea from soils. *ISME J* 2014;**8**:1115–1125. https://doi.org/10.1038/ismej.2013.205

17. Santoro AE, Buchwald C, McIlvin MR, et al. Isotopic signature of N_2_O produced by marine ammonia-oxidizing archaea. *Science* 2011;**333**:1282–1285. https://doi.org/10.1126/science.1208239

18. Sutka RL, Ostrom NE, Ostrom PH, et al. Distinguishing nitrous oxide production from nitrification and denitrification on the basis of isotopomer abundances. *Appl Environ Microbiol* 2006;**72**:638–644. https://doi.org/10.1128/AEM.72.1.638-644.2006

19. Yoshida N, Hattori A, Saino T, et al. ^15^N/^14^N ratio of dissolved N_2_O in the Eastern Tropical Pacific Ocean. *Nature* 1984;**307**:442–444. https://doi.org/10.1038/307442a0

20. Casciotti KL, Sigman DM, Hastings MG, et al. Measurement of the oxygen isotopic composition of nitrate in seawater and freshwater using the denitrifier method. *Anal Chem* 2002;**74**:4905–4912. https://doi.org/10.1021/ac020113w

21. Toyoda S, Mutobe H, Yamagishi H, et al. Fractionation of N_2_O isotopomers during production by denitrifier. *Soil Biology and Biochemistry* 2005;**37**:1535–1545. https://doi.org/10.1016/j.soilbio.2005.01.009

22. Wahlen M, Yoshinari T. Oxygen isotope ratios in N_2_O from different environments. *Nature* 1985;**313**:780–782. https://doi.org/10.1038/313780a0

23. Ostrom NE, Pitt A, Sutka R, et al. Isotopologue effects during N_2_O reduction in soils and in pure cultures of denitrifiers. *J Geophys Res-Biogeosci* 2007;**112**:G02005. https://doi.org/10.1029/2006JG000287

24. Walters W, Hyde ER, Berg-Lyons D, et al. Improved bacterial 16S rRNA gene (V4 and V4-5) and Fungal internal transcribed spacer marker gene primers for microbial community surveys. *mSystems* 2015;**1**:e00009–15. https://doi.org/10.1128/msystems.00009-15

25. Francis CA, Roberts KJ, Beman JM, et al. Ubiquity and diversity of ammonia-oxidizing archaea in water columns and sediments of the ocean. *Proc Natl Acad Sci U S A* 2005;**102**:14683–14688. https://doi.org/10.1073/pnas.0506625102

26. Rotthauwe JH, Witzel KP, Liesack W. The ammonia monooxygenase structural gene amoA as a functional marker: Molecular fine-scale analysis of natural ammonia-oxidizing populations. *Appl Environ Microbiol* 1997;**63**:4704–4712. https://doi.org/10.1128/AEM.63.12.4704-4712.1997

27. Alonso-Sáez L, Waller AS, Mende DR, et al. Role for urea in nitrification by polar marine Archaea. *Proceedings of the National Academy of Sciences* 2012;**109**:17989–17994. https://doi.org/10.1073/pnas.1201914109

**Supplementary Tables are available as separate Excel files:**

**Supplementary Table S1.** Depth profile of the environmental parameter, ammonia oxidation, urea oxidation and N_2_O production rate.

**Supplementary Table S2.** Isotopic fractionation or SP values during N_2_O production and consumption.

**Supplementary Table S3.** Contribution of N_2_O production estimated based on isotope mixing and fractionation models.

**Supplementary Table S4.** NCycDB output showing number of reads assigned to each gene per metagenome

**Supplementary Table S5.** Linear discriminate analysis comparing the abundance of protein-coding sequences encoding nitrogen-cycling proteins (NCycDB).

**Supplementary Table S6.** Spearman’s correlations between rates and taxon relative abundances

**Supplementary Table S7.** Assemblage information of four WTP MAGs.

**Supplementary Table S8.** Characteristics of primer sets, including the 16S rRNA and qPCR, used for the amplification and analysis of genes involved in nitrification processes.

**Supplementary Table S9.** Metadata of TARA Oceans and estuary metagenome-assembled microbial genomes analysed in this study.
